# Single-cell multiomics analysis reveals regulatory programs in clear cell renal cell carcinoma

**DOI:** 10.1038/s41421-022-00415-0

**Published:** 2022-07-19

**Authors:** Zhilin Long, Chengfang Sun, Min Tang, Yin Wang, Jiayan Ma, Jichuan Yu, Jingchao Wei, Jianzhu Ma, Bohan Wang, Qi Xie, Jiaming Wen

**Affiliations:** 1 College of Life Sciences, Zhejiang University, Hangzhou, Zhejiang China; 2 Westlake Laboratory of Life Sciences and Biomedicine, Hangzhou, Zhejiang China; 3 Key Laboratory of Growth Regulation and Translational Research of Zhejiang Province, School of Life Sciences, Westlake University, Hangzhou, Zhejiang China; 4 Institute of Basic Medical Sciences, Westlake Institute for Advanced Study, Hangzhou, Zhejiang China; 5 Department of Urology, The Second Affiliated Hospital, School of Medicine, Zhejiang University, Hangzhou, Zhejiang China; 6 Department of NanoEngineering, University of California San Diego, La Jolla, CA USA; 7 Institute for Artificial Intelligence, Peking University, Beijing, China

**Keywords:** Tumour heterogeneity, Epigenetics

## Abstract

The clear cell renal cell carcinoma (ccRCC) microenvironment consists of many different cell types and structural components that play critical roles in cancer progression and drug resistance, but the cellular architecture and underlying gene regulatory features of ccRCC have not been fully characterized. Here, we applied single-cell RNA sequencing (scRNA-seq) and single-cell assay for transposase-accessible chromatin sequencing (scATAC-seq) to generate transcriptional and epigenomic landscapes of ccRCC. We identified tumor cell-specific regulatory programs mediated by four key transcription factors (TFs) (*HOXC5*, *VENTX*, *ISL1*, and *OTP*), and these TFs have prognostic significance in The Cancer Genome Atlas (TCGA) database. Targeting these TFs via short hairpin RNAs (shRNAs) or small molecule inhibitors decreased tumor cell proliferation. We next performed an integrative analysis of chromatin accessibility and gene expression for CD8^+^ T cells and macrophages to reveal the different regulatory elements in their subgroups. Furthermore, we delineated the intercellular communications mediated by ligand–receptor interactions within the tumor microenvironment. Taken together, our multiomics approach further clarifies the cellular heterogeneity of ccRCC and identifies potential therapeutic targets.

## Introduction

Clear cell renal carcinoma (ccRCC) is the most common and aggressive histological subtype of renal cell carcinoma^1,2^. More than one-third of ccRCC patients relapse and develop metastases after surgery. The prognosis for metastatic ccRCC patients is poor, with a 5-year survival rate of 10%^3^, emphasizing the need to understand the underlying cellular and molecular mechanisms to facilitate the discovery of biomarkers and guide clinical intervention. The efficacy of current ccRCC clinical treatment modalities, including conventional chemotherapy, targeted therapy, and immunotherapy, is limited by tumor heterogeneity^2,4^.

Previously, large-scale genomic studies revealed many essential genome mutations that drive tumor progression and contribute to clinical treatment^5–7^. A common mutation is the inactivation of the *VHL* gene, leading to the stabilization of oncogenic hypoxia-inducible factor proteins (*HIF1α*, *HIF2α*). Other frequent mutations in chromatin remodeling genes, such as *PBRM1*, *BAP1*, and *SETD2*, are necessary for tumorigenesis and tumor development^7,8^. However, tumors exist in a highly heterogeneous microenvironment that includes many different cell types, so bulk sequencing is not suitable for delineating tumor characteristics at the cellular level. Single-cell isolation and barcoding technologies have enabled us to investigate cellular heterogeneity at a single-cell resolution to discern the role of different cell populations whose gene expression patterns may be masked or diluted in bulk sequencing^9,10^. ScRNA-seq has been used to comprehensively characterize the cellular composition and transcriptional states of ccRCC, revealing its origin and intratumoral heterogeneity^11,12^. Comparative scRNA-seq analyses of different conditions (e.g., before and after treatment, in disease stages, and at sampling sites) revealed multiple cell populations associated with immunotherapy resistance and a poor prognosis in ccRCC patients^13–15^. Although significant progress has been made toward delineating changes in the transcriptional expression of various cell types during tumor development and clinical treatment, it remains unclear how cis-acting DNA elements (e.g., enhancers and promoters) and trans-acting factors (e.g., transcription factors (TFs)) regulate these changes within the microenvironment.

ScATAC-seq identifies accessible chromatin regions by Tn5 transposase-mediated tagmentation and captures active DNA regulatory elements at a single-cell resolution^16^. Similar to bulk ATAC-seq, this approach can capture multiple types of gene regulatory information; for example, it can identify genome-wide *cis*-elements and indicate inference with TFs binding and activity. ScATAC-seq has been widely used to delineate the differentiation trajectory of developmental lineages and reveal key regulatory elements^17,18^. Multidimensional data, such as DNA and RNA sequencing data, provide different perspectives to investigate biological phenomena and deepen our understanding of tumor pathogenesis and progression. Recent studies integrating scRNA-seq and scATAC-seq data on healthy kidneys have depicted the transcriptional and chromatin accessibility landscape and identified key celltype-specific TFs that play crucial roles in kidney development^19,20^. Resolving ccRCC heterogeneity with multidimensional information at the single-cell level can provide new insights for exploring tumor regulatory mechanisms and identifying potential therapeutic targets.

Here, we performed scRNA-seq and scATAC-seq on ccRCC primary tumor tissues to identify the key regulatory molecules that mediate tumor development and manipulate the function of immune cells. Our multiomics analysis revealed a tumor-specific regulatory signature and multiple TFs associated with immune cell functional states. Our work provides important insights into dissecting the tumor heterogeneity of ccRCC using single-cell multiomics data.

## Results

### Single-cell transcriptional and chromatin accessibility profiling in ccRCC

**Fig. 1:**
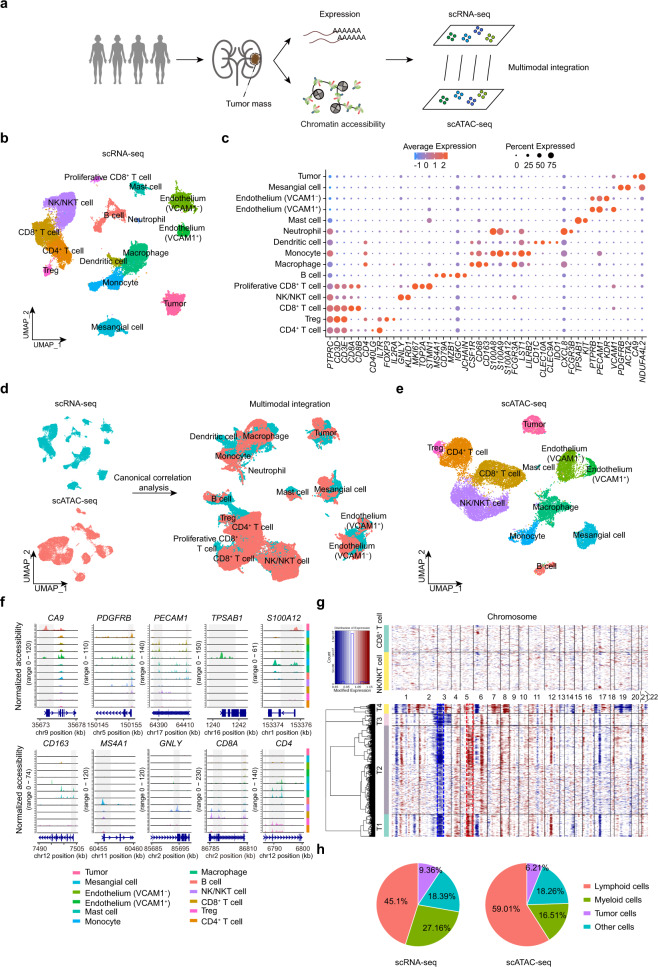
Single-cell transcriptional and chromatin accessibility profiling of human ccRCC. **a** Schematic of multiomics profiling of chromatin accessibility and transcription in ccRCC using scRNA-seq and scATAC-seq. **b** scRNA-seq UMAP projection of 38,097 single cells from four ccRCC samples. **c** Dot plot showing the gene expression patterns of cell-type marker genes in the scRNA-seq data. **d** Cross-platform linkage of scATAC-seq cells with scRNA-seq cells. **e** scATAC-seq UMAP projection of 21,272 single cells from three ccRCC samples. **f** Normalized chromatin accessibility profiles for each cell type at canonical marker genes. The promoter region is highlighted in gray with the gene model and chromosome position shown below. **g** Heatmap of CNV signals normalized against the “normal” cluster shown in the top panel (CD8^+^ T cells and NK/NKT cells) for CNV changes by chromosome (columns) within individual cells (rows). All cells in the tumor population (bottom panel) exhibited chromosome 3p loss (blue dotted frame) or chromosome 5q gain (red dotted frame), which are classic genomic features of ccRCC. **h** Pie charts of the proportion of each class in the scRNA-seq and scATAC-seq dataset.

To systematically dissect the heterogenous architecture of ccRCC tumors, we performed paired scRNA-seq and scATAC-seq on three patients and scRNA-seq alone on one additional patient (Fig. 1a). Selected patients ranged in age from 33 to 75 years old and included both women (*n* = 2) and men (*n* = 2) (Supplementary Table S1). All samples had *VHL* gene mutations (Supplementary Fig. S1a). Other common mutations, such as the histone deubiquitinase *BAP1*^8^ and SWI/SNF component *ARID1B*^21^, were also detected sporadically in these samples. After quality-control filtering, a total of 38,097 cells from scRNA-seq and 21,272 cells from scATAC-seq were retained for the following analysis (Supplementary Fig. S1b–e). Using canonical markers, we identified 15 cell types in the scRNA-seq dataset (Fig. 1b, c and Supplementary Tables S2, S3). For our scATAC-seq data, we calculated the prediction scores by Seurat’s label-transfer algorithm and annotated cell clusters in a supervised manner (Fig. 1d and Supplementary Fig. S1f, g). In parallel, we inspected the chromatin accessibility at the promoter regions for known marker genes and calculated their activity scores for assigning cell identities (Supplementary Fig. S1h). Comparing the annotation results between these two annotation strategies, we found that most cell types were present in both datasets, which supported that scATAC-seq is comparable to scRNA-seq in the detection and annotation of cell types (Supplementary Fig. S1i). Finally, we identified 12 cell types in the scATAC-seq dataset by combining these two annotation results (Fig. 1e, f).

We identified five major lymphocyte populations: CD4^+^ (*CD4*, *IL7R*, *CD3D*, *CD3E*) and CD8^+^ T cells (*CD8A*, *CD8B*, *CD3D*, *CD3E*), Treg (*FOXP3*, *IL2RA*), natural killer (NK)/natural killer T (NKT) cells (*KLRD1*, *GNLY*), B cells (*MS4A1*/*CD20*, *CD79A*) and multiple myeloid subsets after integration of scRNA-seq data and scATAC-seq data, including macrophages (*CSF1R*, *CD68*, *CD163*), monocytes (*S100A12*, *FCGR3A*/*CD16*), and mast cells (*TPSAB1*, *KIT*) (Fig. 1c, f and Supplementary Fig. S1j). We also identified several nonimmune cell types, including endothelial cells (*PECAM1*, *PTPRB*) and mesangial cells (*PDGFRB*, *ACTA2*). Notably, we found two different endothelial subpopulations: one specifically expressed vascular cell adhesion molecule 1 (VCAM1), while the other did not express *VCAM1* but had enriched kinase insert domain receptor (KDR). Similar to a previous study^11^, we found that the VCAM1^−^ endothelial cells highly expressed genes regulating endothelial cell proliferation and vasculature development, whereas the VCAM1^+^ endothelial cells highly expressed genes regulating immune cell chemotaxis and migration (Supplementary Fig. S1k). Next, we identified the tumor cells by detecting both the canonical marker *CA9*^11,22^ and classic copy number variations (CNVs) (chromosome 3p loss or chromosome 5q gain)^8^ (Fig. 1c, f, g). In the entire tumor microenvironment (TME) of ccRCC, we found that immune cells were the most numerous cell population, accounting for more than 70% of the total cells, while tumor cells accounted for <10% of the population (9.36% from scRNA-seq data; 6.2% from scATAC-seq data) (Fig. 1h). Moreover, we found that the abundance of cell types varied across samples, suggesting a considerable level of tumor heterogeneity (Supplementary Fig. S1l). In summary, our integrated scRNA-seq and scATAC-seq multiomics approach revealed the heterogeneous cellular composition of ccRCC.

### Tumor-specific regulatory elements in ccRCC

**Fig. 2:**
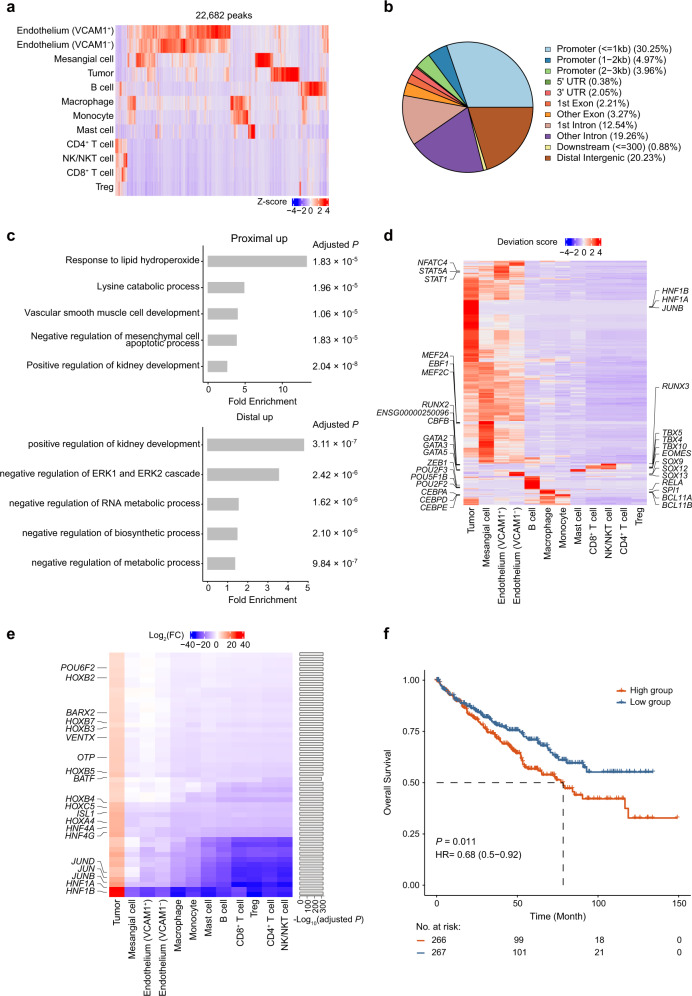
Identification of tumor-specific regulatory elements. **a** Heatmap of the Z-score normalized accessibilities of 22,682 scATAC-seq differential peaks for each cell type. **b** Pie plot of genomic annotations for scATAC-seq differential peaks. **c** Gene ontology term enrichment analysis for distal (intergenic region) and proximal (promoter and gene body) upregulated DARs in tumor cells. The Benjamini-Hochberg adjusted *P* values are shown on the right side of the plots. **d** Heatmap representation of chromVAR bias-corrected deviation scores for the differential TF motifs of each cell type. The top TF motifs of each cell type are indicated on both sides of the plot. **e** Heatmap of the log_2_(FC) values for tumor-specific TFs identified by our filtering strategy. The right bar plot shows the Bonferroni-adjusted *P* values. **f** Kaplan–Meier overall survival curves of TCGA-KIRC patients grouped by the average expression of tumor-specific TFs (with the median value as the threshold). HR hazard ratio.

To investigate the differences in chromatin accessibility among all cell types, we first identified a total of 212,326 peaks in our scATAC-seq data by using MACS2^23^ and found that ~10.6% (22,682 unique peaks) of them exhibited significant differences among cell types; these sites were defined as differentially accessible chromatin regions (DARs) (adjusted *P* < 0.05 and log_2_(fold change (FC)) > 0.25) (Fig. 2a). Approximately 17.3% (mean proportion = 0.173 ± 0.067) of DARs were closely associated with differentially expressed genes (DEGs) in their respective cell types (Supplementary Table S4). Overall, endothelium and mesangial cells had the most DARs, followed by tumor cells (Supplementary Fig. S2a). Notably, lymphoid lineage populations, except B cells, had fewer DARs than myeloid cell populations. The majority of DARs were located in the promoter and intronic regions of the genome, and the distribution of DARs was relatively conserved across cell types (Fig. 2b and Supplementary Fig. S2b). In addition, we found that both DARs and DEGs of tumor cells were significantly enriched in metabolism-related biological processes (Fig. 2c and Supplementary Fig. S2c). Next, we applied chromVAR^24^ to infer TF motif activity in our scATAC-seq data. Hierarchical clustering of the bias-corrected deviation scores for the differential TFs revealed shared and unique regulatory elements across cell types (Fig. 2d and Supplementary Table S5). The identification of distinct lineage-specific TFs further supported our assignment of cell identities. For example, T-Box (e.g., *EOMES* and *TBX5*) TFs were exclusively enriched in NK/NKT and CD8^+^ T cell populations^25,26^. Within myeloid cells, *SPI1*^27^ TF were enriched in macrophages, CEBP TF family^28^ exhibited high activity in monocytes, while the GATA2^29^ TF was specifically enriched in mast cells. Interestingly, two endothelial subpopulations exhibited differences in TF enrichment: the VCAM1^−^ endothelial cells showed high activity of SOX TF family, while *STAT1* and *NFATC4* were enriched in the VCAM1^+^ endothelial cells.

To uncover the pivotal TFs participating in tumorigenesis and development, we designed a filtering strategy to identify highly specific TFs enriched in tumor cells. First, we calculated the standard deviation of chromVAR bias-corrected scores among all cell types for each TF and filtered out the TFs with low variability by the median absolute deviation method^30^. Then, we selected the TFs highly enriched exclusively in tumor cells (log_2_(FC) > 4 in tumor cells and log_2_(FC) < 1 in any other cell types; adjusted *P* < 0.0001) and identified 49 TFs with higher activity scores in tumor cells than in any other cell types (Fig. 2e and Supplementary Table S6). Hepatocyte nuclear factor 1 (*HNF1A, HNF1B*), a family of tissue-restricted transcription regulators associated with kidney developmental disorders^31^, had the highest activity scores and were highly expressed in tumor cells specifically (Supplementary Fig. S2d). Multiple HOX family TF genes were also highly enriched in tumor cells and have been reported to play crucial roles in numerous tumor processes, including angiogenesis and oncogenesis^32,33^. We then examined the association of these tumor-specific TFs with the prognosis of patients in The Cancer Genome Atlas kidney renal clear cell carcinoma (TCGA-KIRC) cohort. Patients with high average expression of tumor-specific TFs had shorter overall survival and disease-free survival than the patients with low average expression (Fig. 2f and Supplementary Fig. S2e).

**Fig. 3:**
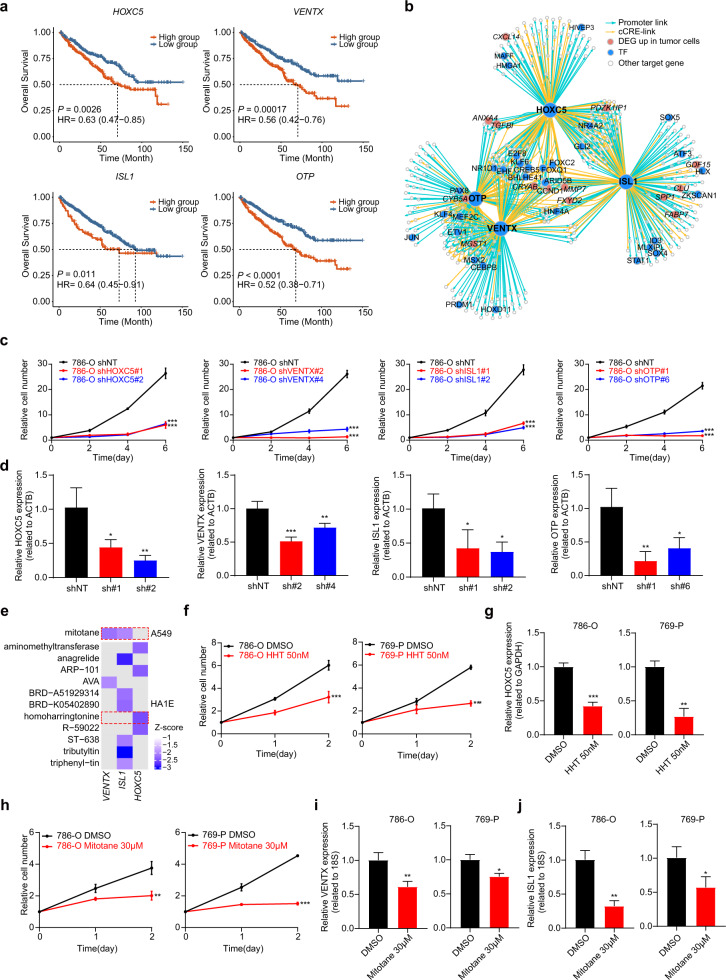
Characterization of the four tumor-specific TFs. **a** Kaplan–Meier analyses of overall survival in TCGA-KIRC patients separated by *HOXC5*, *VENTX*, *ISL1*, and *OTP* expression (with the median value as the threshold) individually. HR, hazard ratio. **b** TF regulatory network showing the candidate target genes for the following TFs: *HOXC5*, *VENTX*, *ISL1*, and *OTP* in tumor cells. **c** The effects of sh-*HOXC5*, *VENTX*, *ISL1*, *OTP*, and vector on cell proliferation were determined by a cell proliferation assay in the 786-O cell line. **d** *HOXC5*, *VENTX*, *ISL1*, and *OTP* mRNA expression were significantly downregulated in the 786-O cell line. Significance was determined by two-way ANOVA. **e** Heatmap showing the degree of decrease in expression levels of *VENTX*, *ISL1*, and *HOXC5* after drug treatment. **f** Cell proliferation was assessed over a 2-day time course after treatment with DMSO or HHT in the 786-O (top) and 769-P (bottom) ccRCC cell lines. Significance was determined by multiple *t-*tests. **g** The mRNA levels of *HOXC5* after treatment with DMSO or HHT in the 786-O (left) and 769-P (right) ccRCC cell lines were measured using qPCR. Significance was determined by Student’s *t*-test. **h** Cell proliferation was assessed over a 2-day time course after treatment with DMSO or mitotane in the 786-O (top) and 769-P (bottom) ccRCC cell lines. Significance was determined by multiple *t*-tests. **i** The mRNA levels of *VENTX* after treatment with DMSO or mitotane in the 786-O (left) and 769-P (right) ccRCC cell lines were measured using qPCR. Significance was determined by Student’s *t*-test. **j** The mRNA levels of *ISL1* after treatment with DMSO or mitotane in the 786-O (left) and 769-P (right) ccRCC cell lines were measured using qPCR. Significance was determined by Student’s *t*-test. For all statistical tests, *P < 0.05; **P < 0.01; ***P < 0.001.

To further investigate and verify the biological functions of these TFs in ccRCC, we selected four TFs (*HOXC5*, *VENTX*, *ISL1*, and *OTP*) whose expression levels were significantly associated with worse overall survival in the TCGA-KIRC dataset, and binding sites were located in accessible chromatin regions, which were specific for kidney cancer and identified by ATAC-seq^34^ (Fig. 3a and Supplementary Fig. S3a). We identified their target genes whose promoters or linked candidate *cis*-regulatory elements (cCREs) were accessible and contained the TF-binding motif in tumor cells. Then, we constructed TF regulatory networks to gain further insights into TF-mediated gene regulation in tumor cells. Within these networks, we found that these four TFs regulated multiple tumor-specific genes, such as *FXYD2* and *CRYAB*, with a significant increase in expression in tumor cells (Fig. 3b and Supplementary Table S3). Moreover, we found that the target genes of these TFs were significantly enriched in hypoxia and cell proliferation signaling pathways (Supplementary Fig. S3b). For each TF, we calculated the target gene scores for each cell type and found that the scores in tumor cells were significantly higher than in any other cell type (Supplementary Fig. S3c). Similar phenomena were observed in the other three previous scRNA-seq datasets of ccRCC^11,13,14^ (Supplementary Fig. S3d–f). More importantly, we found that knocking down these TFs significantly reduced tumor cell proliferation and increased cell death (Fig. 3c, d and Supplementary Fig. S3g, h). We further confirmed that knocking down two TFs (*HOXC5* and *ISL1*) strongly reduced tumor growth in the xenograft mouse models (Supplementary Fig. S3i–k). To find potential drugs for targeting these TFs, we used the LINCS consortium^35^, which included 19,811 small molecule compound-perturbed profiles, to interrogate the effects of drugs on gene expression. With this information, we found two candidate drugs (homoharringtonine (HHT) and mitotane) approved by the FDA could significantly decrease the expression level of *HOXC5*, *ISL1*, and *VENTX* (Fig. 3e). *OTP* was not included in the drug perturbation expression profile and thus was not subjected to subsequent analysis. As we expected, both of these drugs significantly decreased the proliferation rate of tumor cells and the expression levels of *HOXC5*, *ISL1*, and *VENTX* in two renal cancer cell lines (Fig. 3f–j). Collectively, we identified tumor-specific regulatory elements with chromosome accessibility signals captured by scATAC-seq and verified their roles in promoting tumor growth.

### Malignant transcriptional programs within ccRCC

**Fig. 4:**
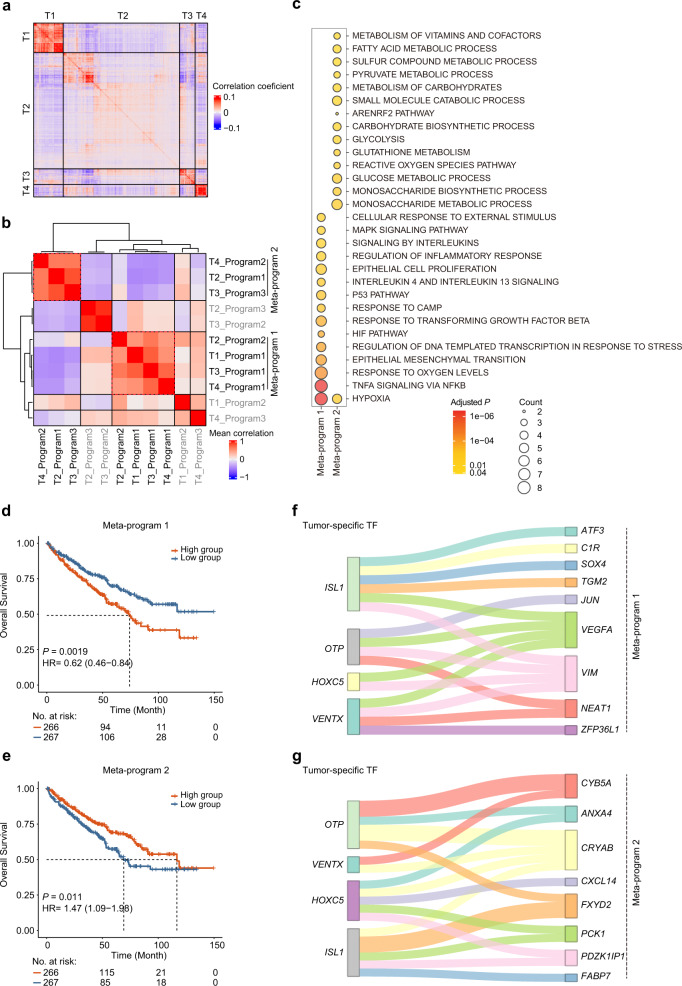
Deciphering malignant transcriptional programs within ccRCC. **a** Pairwise correlations between the expression profiles of four scRNA-seq samples (3564 cells). **b** Heatmap of average correlations across four samples between pairs of programs. **c** Dot plot showing significantly enriched pathways for signature genes of each meta-program. The color shade of the dot indicates the *P* value after FDR correction (the redder, the smaller adjusted *P* value), and the size of the dot represents the number of genes in the indicated pathway (the larger, the more genes included). **d**, **e** The Kaplan–Meier overall survival curves of TCGA-KIRC patients grouped by the average gene expression of meta-program 1 and meta-program 2 (with the median as the threshold). The *P* value was calculated by the log-rank test. HR, hazard ratio. **f**, **g** Sankey diagrams showing the regulatory relationship between the four tumor-specific TFs and genes within meta-program 1 and meta-program 2.

To explore how expression states vary among different malignant cells within ccRCC, we focused on 3564 tumor cells from four samples (Supplementary Fig. S4a). Pairwise correlation analysis revealed multiple distinct transcriptional states that were consistently present within these tumors (Fig. 4a). To interrogate tumor heterogeneity information more precisely, we applied nonnegative matrix factorization (NMF) to define underlying transcriptional programs consisting of coexpressed genes. NMF is a matrix factorization method and can extract meaningful features from noisy or complex data in a direct, unbiased manner^36^. Due to its excellent performance in dimensionality reduction and data interpretability, NMF is widely used to identify key subclasses and latent biological processes in the scRNA-seq dataset^37,38^. With this method, we successfully extracted a total of 11 intratumor programs among these samples (Supplementary Fig. S4b, c). Subsequently, we refined these programs by hierarchical clustering and identified two meta-programs that included highly similar programs across the four samples (Fig. 4b and Supplementary Fig. S4d, Table S7). Meta-program 1 was present in all samples, while meta-program 2 was present in three samples. The cluster of programs that covered less than half the samples was not subjected to subsequent analysis. These two meta-programs exhibited a relatively low correlation (*r* = −0.2), suggesting the different roles in tumors (Supplementary Fig. S4e). Next, we investigated the biological functions of these meta-programs using pathway enrichment analysis. The genes of meta-program 1 were significantly enriched in stress-related pathways, such as hypoxia and the MAPK signaling pathway, while the genes of meta-program 2 were mainly involved in metabolic-related biological processes, such as glycolysis and monosaccharide metabolism (Fig. 4c). Furthermore, we examined the association between the average gene expression of each meta-program and the prognosis of patients in the TCGA-KIRC cohort. Patients with high expression of meta-program 1 had worse overall survival than patients with low expression, while patients with high expression of meta-program 2 were associated with better overall survival (Fig. 4d, e).

We further interrogated the key regulatory molecules for these two meta-programs. With the previous strategy for the identification of TF target genes, we found that more than two-thirds of the genes in each meta-program can be regulated by numerous TFs (Supplementary Fig. S4f). Multiple TFs, such as *ZNF263* and *SP1*, showed similar and strong regulatory capabilities in different meta-programs. We noticed that *VEGFA* and *SMIM24* were the cardinal coregulatory target genes of these TFs for meta-program 1 and meta-program 2, respectively (Supplementary Fig. S4g, h). We further explored the regulatory relationship between meta-programs and four tumor-specific TFs identified previously. Notably, we also found that the four TFs all exhibited a regulatory relationship with *VEGFA* in meta-program 1 (Fig. 4f). In meta-program 2, *CRYAB*, the second most significant DEG in tumor cells, was regulated by all four TFs (Fig. 4g). The most significant DEG in tumor cells, *FXYD2*, was regulated by both *ISL1* and *OTP*. Overall, we discovered two major transcriptome programs within ccRCC tumor cells and further identified the possible regulatory elements of these two programs.

### CD8^+^ T cell clustering and state analysis in ccRCC

**Fig. 5:**
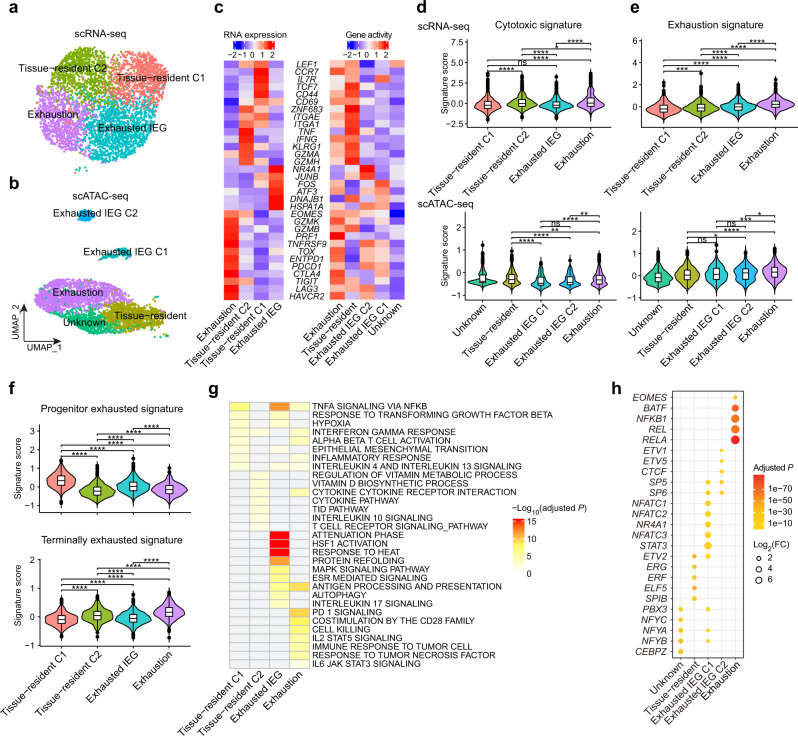
Characterization of CD8^+^ T cells in ccRCC. **a**, **b** Subclustering of CD8^+^ T cells on the UMAP plots of the scRNA-seq and scATAC-seq datasets. **c** Heatmap of CD8^+^ T cell lineage and functional markers provided phenotypic information for individual CD8^+^ T cell clusters in the scRNA-seq (left) and scATAC-seq (right) data. **d**, **e** Violin plots showing the signature scores of cytotoxic and exhaustion gene sets for each CD8^+^ T cell cluster in the scRNA-seq (top) and scATAC-seq data (bottom). Signature scores for each cell were calculated by the VISION method. Two-sided Wilcoxon test. ns, no significance; *P < 0.05; **P < 0.01; ***P < 0.001; ****P < 0.0001. **f** Violin plots showing the progenitor and terminally exhausted signature scores for each CD8^+^ T cell cluster in the scRNA-seq data. Signature scores for each cell were calculated by the VISION method. Two-sided Wilcoxon test. ns, no significance; *P < 0.05; **P < 0.01; ***P < 0.001; ****P < 0.0001. **g** Heatmap of significantly enriched pathways for DEGs of each CD8^+^ T cell cluster in the scRNA-seq data. The darker the red color is, the smaller the adjusted *P* value. **h** Dot plot showing the top enriched TFs within each CD8^+^ T cell subpopulation individually.

CD8^+^ T lymphocytes play crucial roles in inhibiting tumor progression. To better elucidate the heterogeneity of tumor-infiltrating CD8^+^ T cells, we further investigated CD8^+^ T cell subpopulations in both scRNA-seq and scATAC-seq datasets. We focused on the major subpopulations and removed clusters with fewer than 100 cells (Supplementary Fig. S5a–f). Finally, we identified four major clusters in the scRNA-seq dataset and five major clusters in the scATAC-seq dataset (Fig. 5a, b). Tissue-resident CD8^+^ T cells were identified in both scRNA-seq and scATAC-seq datasets with high gene expression or activity of tissue-resident markers (*CD69*, *ZNF683*/*Hobit*, *ITGAE*/*CD103*, and *ITGA1*/*CD49A*)^39–41^ (Fig. 5c). Interestingly, tissue-resident CD8^+^ T cells included two clusters in the scRNA-seq data: one cluster (tissue-resident C2) exhibited high expression of effector molecules (*TNF*, *IFNG*, and *GZMA*), and the other (tissue-resident C1) was highly expressed naive/memory genes (*IL7R*, *CCR7*, and *TCF7*). We identified the exhausted CD8^+^ T cells based on the expression of exhaustion markers (*PDCD1* and *TOX*^42^) (Fig. 5c and Supplementary Fig. S5g). The cluster (“exhausted immediate-early genes (exhausted IEG)”) was also characterized by the high expression of genes induced early after activation (e.g., *HSPA1A*, *DNAJB1*, *JUNB*, and *ATF3*), which is consistent with a recent study^15^. Notably, we identified two exhausted IEG clusters in the scATAC-seq dataset and exhibited different gene activities in T cell inhibitory genes (*CTLA4*, *LAG3*, and *HAVCR2*/*TIM3*).

We further investigated the functional properties of these CD8^+^ T cell subpopulations using multiple functional gene sets^13,43^ and the VISION method^44^ (Supplementary Table S2). The exhaustion cluster presented the highest cytotoxic, exhaustion, and terminal differentiation signature scores (Fig. 5d, e and Supplementary Fig. S5h). Exhausted IEG cells had higher cell stress signature scores than other cells (Supplementary Fig. S5i). Recent studies have reported that progenitor exhausted T cells control tumors more effectively than terminally exhausted T cells and respond better to anti-PD1 therapy^43,45^. Thus, we assessed progenitor and terminally exhausted signatures and found that the tissue-resident C1 cluster had the highest progenitor exhausted signature scores and the lowest terminally exhausted signature scores (Fig. 5f), indicating an important role in tumor immunotherapy. In parallel, we performed pathway enrichment analysis for DEGs (adjusted *P* < 0.05 and log_2_(FC) > 0.5) of each cluster in our scRNA-seq dataset. We found that two tissue-resident clusters enriched distinct pathways: the tissue-resident C1 cluster was significantly associated with the inflammatory response and the tissue-resident C2 cluster was enriched in cytokine and vitamin metabolic pathways (Fig. 5g). Multiple T cell exhaustion-related pathways, such as PD-1, IL2-STAT5^46^, and IL6-JAK-STAT3^47^ signaling, were significantly enriched in the exhaustion cluster. Furthermore, we found that the average expression of DEGs in the exhaustion cluster was associated with worse overall survival in the TCGA-KIRC and CheckMate-025 cohorts, a randomized phase III trial of nivolumab (anti-PD-1) treatment in ccRCC^48^ (Supplementary Fig. S5j). Next, we examined the regulatory elements in these CD8^+^ T clusters. *EOMES* and *BATF* implicating in CD8^+^ T cell exhaustion^49,50^ were highly enriched in the exhaustion cluster (Fig. 5h). Multiple Rel/NF-κB family TFs, such as *RELA* and *NFKB1*, were also enriched in the exhaustion cluster. Interestingly, we found that two exhausted IEG clusters were regulated by different TFs.

### Macrophage clustering and state analysis in ccRCC

**Fig. 6:**
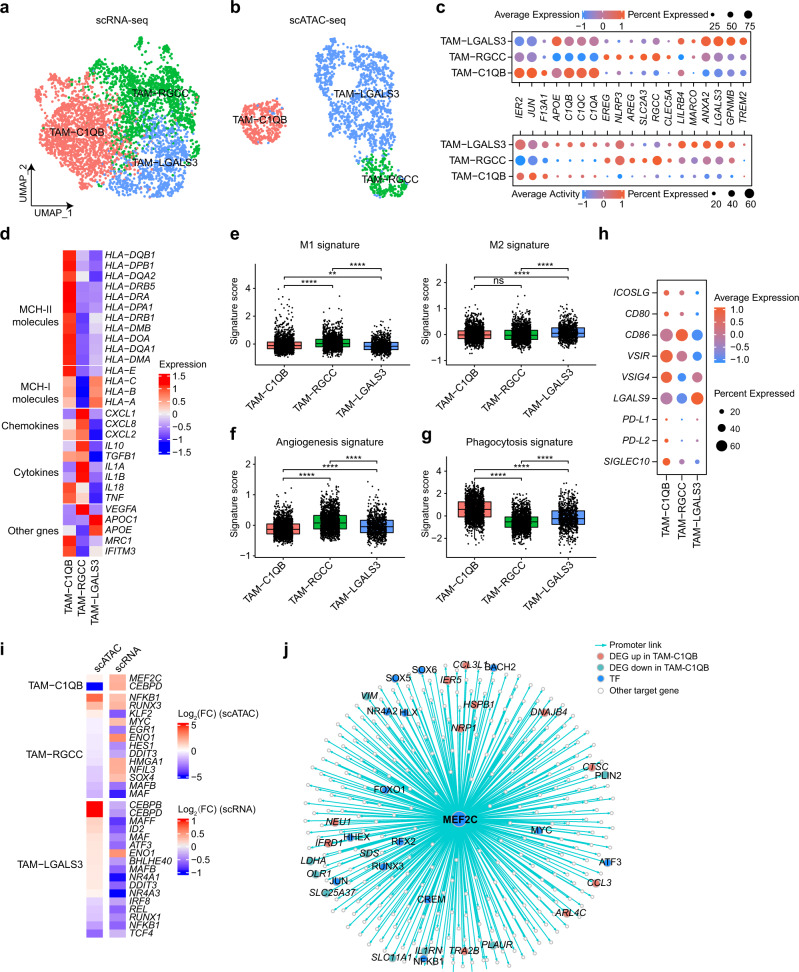
Characterization of TAMs in ccRCC. **a**, **b** Subclustering of TAMs on the UMAP plots of the scRNA-seq and scATAC-seq datasets. **c** Dot plot showing the expression levels and activity scores for known phenotypic markers in each TAM cluster. Dot size indicates the fraction of expressing cells, colored based on normalized expression or activity. **d** Heatmap showing the expression of MHC molecules, chemokines, cytokines, and other related genes in each TAM cluster. **e** Boxplots showing the M1 and M2 signature scores for each TAM cluster in the scRNA-seq data. The signature score was calculated by the VISION method. Two-sided Wilcoxon test. ns, no significance; *P < 0.05; **P < 0.01; ***P < 0.001; ****P < 0.0001. **f**, **g** Boxplot showing the angiogenesis and phagocytosis signature scores for each TAM cluster in the scRNA-seq data. The signature score was calculated by the VISION method. Two-sided Wilcoxon test. ns, no significance; *P < 0.05; **P < 0.01; ***P < 0.001; ****P < 0.0001. **h** Dot plot showing the expression of the immune costimulatory, checkpoint, and evasion genes for each TAM cluster in the scRNA-seq dataset. **i** Heatmap of log_2_(FC) values for TFs with significant differences both in the scRNA-seq and scATAC-seq datasets. **j** TF regulatory network showing the predicted target genes for *MEF2C* in the TAM-C1QB cluster.

Tumor-associated macrophages (TAMs) are the major population of myeloid cells in tumors and play vital roles in tumorigenesis and drug resistance^51^. To systematically interrogate the heterogeneity of TAMs, we performed recluster analysis similar to CD8^+^ T cells and identified three TAM clusters in both datasets using known phenotypic markers^52^ (Fig. 6a–c and Supplementary Fig. S6a–f). The first cluster (“TAM-C1QB”) expressed complement genes (e.g., *C1QB* and *C1QC*), *APOE*, and early response-related genes (e.g., *IER2* and *JUN*). Moreover, numerous MHC class II molecules were highly expressed in this cluster, suggesting strong antigen presentation ability (Fig. 6d). The second cluster (“TAM-RGCC”) expressed high levels of *RGCC* and proinflammatory genes, such as *NLRP3*^53^, *CLEC5A*, and *IL1A*/*B*. The third cluster (“TAM-LGALS3”) exhibited intermediate expression of *C1QB* and *C1QC* but highly expressed genes involved in alternative (M2) macrophage activation, such as *ANXA2*^54^ and *LGALS3*^55^. In addition, this cluster also expressed immunosuppressive genes, such as *GPNMB*^56^ and *TREM2*^57^, and multiple MHC class I molecules.

To understand the function of these TAM subpopulations, we further examined the expression or activity of multiple functional gene sets^58^ (Supplementary Table S2). We found that the TAM-RGCC cluster exhibited the highest M1 signature scores, followed by the TAM-C1QB cluster (Fig. 6e and Supplementary Fig. 6g). Conversely, the M2 signature was primarily enriched in the TAM-LGALS3 cluster. TAM-RGCC cluster possessed higher angiogenesis signature scores (Fig. 6f and Supplementary Fig. S6h). Notably, the TAM-C1QB cluster showed the highest phagocytosis signature scores in scRNA-seq data, suggesting a high phagocytic activity (Fig. 6g). However, scATAC-seq data did not show a similar trend, maybe due to the sparsity of its data^59^ (Supplementary Fig. S6i). In parallel, we investigated the differences among these subpopulations at the pathway level and found that various pathways related to inflammation (e.g., TNFA signaling via NFKB, and inflammatory response) and phagocytosis (e.g., endocytosis and phagocytosis) were significantly enriched in the TAM-C1QB cluster (Supplementary Fig. S6j). TAM-RGCC cluster exhibited enrichment of angiogenesis-related pathways, while the TAM-LGALS3 cluster exhibited enrichment of lysosome and lipid catabolic processes. Furthermore, we examined the expression of immune checkpoint genes and costimulatory molecules. Multiple costimulatory signals were detected in both TAM-C1QB and TAM-RGCC clusters but not in the TAM-LGALS3 cluster (Fig. 6h). The ligands (*PD-L1*, *PD-L2*, *VSIR/VISTA*^60^, *VSIG4*^61^, and *SIGLEC10*^62^) mediating T cell immune checkpoint were highly expressed in the TAM-C1QB cluster while *LGALS9*^63^ was specifically enriched in the TAM-LGALS3 cluster. Next, we investigated the difference in TF motif activity and found distinct TF regulation programs among the three TAM clusters (Supplementary Fig. S6k). Four TFs (*MEF2C*, *NFKB1*, *RUNX3*, and *ENO1*) showed a substantial increase in both gene expression and activity in the corresponding clusters (Fig. 6i). Previous studies have demonstrated that MEF2C plays vital roles in promoting M1 macrophage polarization and inducing cell death of macrophage^64,65^. We identified *MEF2C* target genes using the same strategy described in the previous section and found that this gene regulated multiple TFs (e.g., *FOXO1*, *NEU1*, and *NRP1*) and chemokines (e.g., *CCL3* and *CCL3L1*) (Fig. 6j) that have been demonstrated to be associated with phagocytosis and angiogenesis in macrophages^66–69^. Moreover, the signature score of *MEF2C* target genes was associated with better survival in both the TCGA-KIRC and CheckMate-025 cohorts (Supplementary Fig. S6l).

### Cell–cell cross-talk in the ccRCC microenvironment

**Fig. 7:**
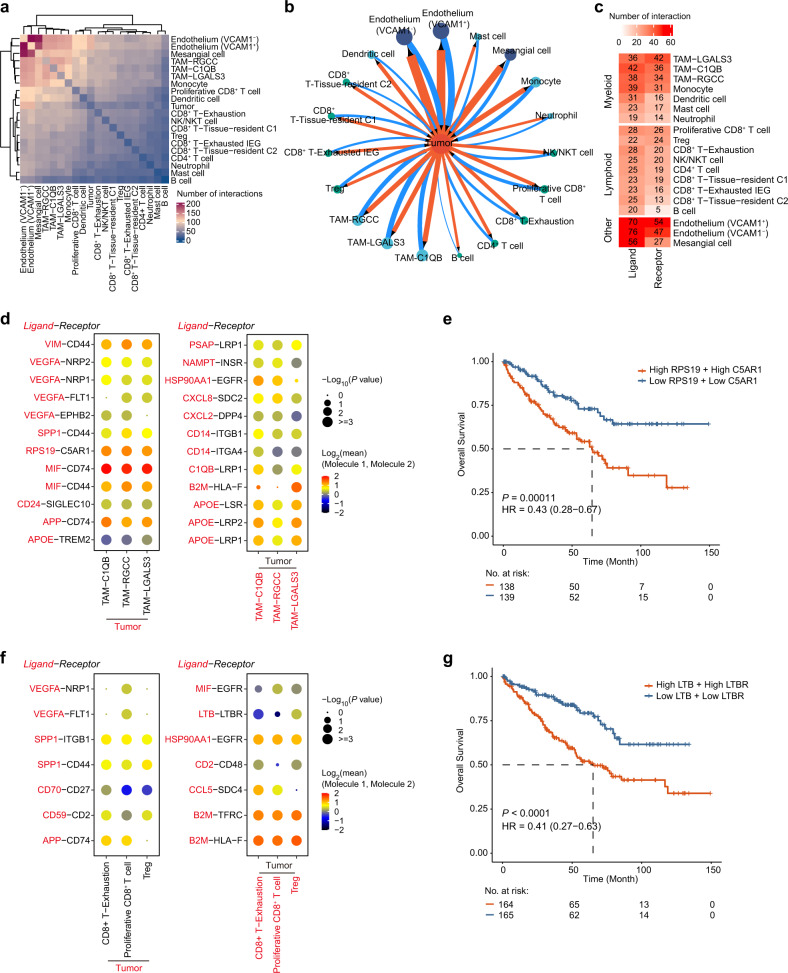
Ligand–receptor-based interaction between tumor and immune cells. **a** Heatmap of the number of significant ligand–receptor interactions between cell types in the scRNA-seq data. **b** The interaction network between the tumor cell and other cell types. The direction of the arrow indicates the cell type expressing the receptor. The wider the edge or larger the dot is, the greater the number of interactions. **c** Heatmap of the number of significant interactions mediated by tumor cells as ligand-expressing and receptor-expressing cells. **d** Dot plots showing the mean interaction strength for selected ligand–receptor pairs between tumor cells and TAM clusters. The left and right plots show that the ligand is expressed in the tumor cells and TAM clusters, respectively. Dot size indicates the *P* value, colored by the average expression level of interacting molecule 1 in cluster 1 and interacting molecule 2 in cluster 2. **e** The Kaplan–Meier overall survival curves of TCGA-KIRC patients grouped by the averaged expression of *RPS19* and *C5AR1* (with the median value as the threshold). HR, hazard ratio. **f** Dot plots showing the mean interaction strength for selected ligand–receptor pairs between tumor cells and three lymphoid subsets. The left and right plots show that the ligand is expressed in the tumor cells and lymphoid subsets, respectively. Dot size indicates the *P* value, colored by the average expression level of interacting molecule 1 in cluster 1 and interacting molecule 2 in cluster 2. **g** Kaplan–Meier overall survival curves of TCGA-KIRC patients grouped by the averaged expression of *LTB* and *LTBR* (with the median value as the threshold).

To demonstrate the intercellular communications within the ccRCC microenvironment, we applied CellPhoneDB, a repository of known ligand–receptor interactions, for data analysis. We found that the endothelial cells had the largest ligand–receptor interaction pairs, while B cells had the fewest (Fig. 7a and Supplementary Fig. S7a). Notably, the TAM populations had the largest number of ligand–receptor interaction pairs compared to other immune cell populations (Supplementary Fig. S7b). Tumor cells are the key cellular component of the entire TME, and the interactions between tumor cells and other cells play crucial roles in tumorigenesis. Therefore, we built an interaction map to investigate the intercellular communications of tumor cells (Fig. 7b). Tumor cells mainly communicated with the endothelium, TAMs, proliferative CD8^+^ T cells, and CD8^+^ exhaustion T cells through multiple ligand–receptor interactions (Fig. 7c and Supplementary Fig. S7c).

The crosstalks between various immune cells (especially macrophages and T cells) and tumor cells play crucial roles in tumor immune escape, which is a hot spot in cancer immunology research. Thus, we selected three cell subpopulations in the myeloid and lymphoid lineages with the highest number of interactions with tumor cells for further study. We found strong interactions between tumor cells and TAM populations mediated by macrophage migration inhibitory factor (MIF) and its receptors (*CD44* and *CD74*), which have been well characterized to play vital roles in tumor progression, angiogenesis, and immune escape in ccRCC^70–72^ (Fig. 7d and Supplementary Fig. S7d). We also uncovered some novel interactions that have not been reported in ccRCC, for example, the ligand–receptor interaction between ribosomal protein S19 (RPS19) and complement C5a receptor 1 (C5AR1), which has been shown to contribute to immunosuppression in the human breast and ovarian cancer TME^73^. Patients with high average expression of *RPS19* and *C5AR1* had a poor prognosis in the TCGA-KIRC dataset (Fig. 7e). Moreover, we identified multiple interactions between tumor cells and three lymphocyte subpopulations (proliferative CD8^+^ T cell, Treg, and CD8^+^ T-exhaustion) (Fig. 7f). Notably, we characterized a Treg-tumor cell-specific interaction mediated by the ligand Lymphotoxin Beta (LTB) and its receptor lymphotoxin beta receptor (LTBR), which is required for fibrosarcoma and hepatocellular carcinoma tumor formation^74,75^. High average expression of *LTB* and *LTBR* was associated with poor clinical outcomes in the TCGA-KIRC dataset, suggesting the translational potential of this finding (Fig. 7g).

## Discussion

Here, we performed integrative scRNA-seq and scATAC-seq to delineate the transcriptional and epigenetic landscape of ccRCC. Our analysis demonstrated that scRNA-seq and scATAC-seq had consistently high cell identification abilities, and their data confirmed and complemented each other. Single-cell multiomics profiling can provide more comprehensive information from different perspectives, enabling us to better dissect cellular compositions and decipher cross-compartment interactions in the TME.

Consistent with several recently reported scRNA-seq studies of ccRCC, we found that tumor cells represent only a small fraction (7.2%) of all cells in ccRCC tissues^11^, suggesting that traditional bulk-level epigenetic sequencing methods, such as ATAC-seq and ChIP-seq, may fail to identify some tumor cell-specific regulatory elements and their networks. In this study, we revealed several pivotal regulatory TFs (*HOXC5*, *ISL1*, *VENTX*, and *OTP*) in tumor cells by integrating scRNA-seq and scATAC-seq data and further experimentally validated their roles in tumor growth. The function of these TFs had been explored in some other types of cancers. For example, ISL1 has been reported as an important regulator and potential therapeutic target for gastric cancer and triple-negative breast cancer^76,77^, while OTP has been characterized as a promising prognostic marker of pulmonary neuroendocrine tumors^78^. Our findings highlight the potential oncogenic roles of these TFs in ccRCC for the first time, but the underlying mechanisms remain to be investigated in future studies.

In addition, multiple hepatocyte nuclear factors (e.g., HNF1A, HNF4A, and HNF4G) we identified in tumor cells have been shown to be specific for proximal tubule cells, which are the original cells of ccRCC^79^. We also adopted a refined clustering strategy on the two major immune cell populations (CD8^+^ T cells and macrophages) in the TME to reveal the TFs that control different functional states of these cells. Multiple NFATC TFs (e.g., NFATC2/NFAT1 and NFATC3/NFAT4) were specifically enriched in the exhausted IEG C1 population and have already been demonstrated to promote exhaustion of T cells and better control immune responses by disrupting its interactions^80,81^. C-Rel (REL) plays a vital role in the activation of Tregs and is a potential target for suppressing Treg function^82^. Interestingly, we found that the REL TF was significantly enriched in the exhaustion T cells, suggesting a potential target for CD8^+^ T cells for future research. Furthermore, we comprehensively delineated the multicellular communities in the TME and identified ligand–receptor pairs, including RPS19-C5AR1 and LTB-LTBR, which could serve as potential therapeutic targets for further studies. Targeting the LTB-LTBR interaction with an agonistic monoclonal antibody (CBE11) inhibited tumor growth and prolonged survival in colorectal cancer xenografts^83^. Therefore, therapeutic interruption of these interactions by antagonist or blocking antibodies may alleviate the immunosuppressive nature of the microenvironment and provide a potential strategy for ccRCC treatment.

In summary, our single-cell multiomics analysis provides a high-resolution transcriptional and epigenomic map of ccRCC and in-depth knowledge of tumor heterogeneity and underlying regulatory factors, facilitating a step forward in rational therapeutic strategy design.

## Materials and methods

### Human specimens

All research activities were pre-approved by The Institutional Research Ethics Committee of The Second Affiliated Hospital, School of Medicine, Zhejiang University (NO. IR2020001463), and Informed consent were obtained for all human participants. Human ccRCC samples were directly obtained from the operating room during nephrectomy and transported in MACS tissue storage solution (#130-100-008; Miltenyi) on ice.

### Tissue dissociation and library preparation

The tumor tissue of each sample was equally divided into two parts for scRNA-seq and scATAC-seq library preparation. For scATAC-seq, fresh nuclei were isolated with 1 mL prepared lysis buffer (10 nM Tris-HCI, 10 mM NaCI, 3 mM MgCl_2_, 0.1% Nonidet^TM^ P40 Substitute (#74385; Sigma-Aldrich)) and incubated on ice for 5 min. The culture was centrifuged at 500× *g* for 5 min at 4 °C and mixed with 1 mL nuclear resuspension buffer (1× PBS, 1% BSA, and 0.2 U/μL RNase Inhibitor). The homogenate was filtered through a 40-μm cell strainer (#H13680-0040; BelArt) and the nuclear concentration was determined using Countess® II FL Automated Cell Counter (#C10228; Thermo Fisher). For scRNA-seq, tissues were cut into 2–4 mm^3^ pieces and dissociated to generate a single-cell suspension. The cell suspension culture was centrifuged at 300× *g* for 30 s and resuspended with an additional 10 mL RPMI 1640 (#10-040-CM; Miltenyi Biotec). The homogenate was filtered through a 70-μm cell strainer (#130-098-462; Miltenyi Biotec) and centrifuged at 300× *g* for 7 min followed by red blood cell lysis with 1 mL 1× Red Blood Cell Lysis Solution (#130-094-183; Miltenyi Biotec). The cell concentration was determined using Countess® II FL Automated Cell Counter. 10× Chromium libraries were prepared according to manufacturer protocol. Three scATAC-seq libraries and four scRNA-seq libraries were obtained using 10× Genomics Chromium Single Cell ATAC v1 chemistry and 10× Genomics Chromium Single Cell 3′ v3 chemistry, respectively. All libraries were sequenced on Illumina NovaSeq 6000 using a paired-end 150 bp protocol.

### scRNA-seq data processing

Raw sequencing data were processed with Cell Ranger (v5.0.0, 10× Genomics) software for demultiplexing, aligning to GRCh38 human reference genome, and generating gene-barcode matrices. Seurat (v4.0.3)^84^ R package was used to perform filtering, normalization, dimensionality reduction, clustering, and differential expression analysis. The following criteria were applied to each sample to remove low-quality cells: gene number between 200 and 6000, UMI count > 1000, and mitochondrial content > 10%. Doublets were predicted by the DoubletFinder (v2.0.3)^85^ algorithm. After filtering, a total of 38,600 cells were left. The batch effect across different samples was eliminated by the Harmony (v1.0)^86^ method. The top 40 harmony embedding was selected by the “ElbowPlot” function and used to perform clustering and visualization. The “FindClusters” function was performed to generate different clustering results with resolutions ranging from 0.2 to 1.2. An appropriate resolution was determined based on cluster stability with clustree (v0.4.3)^87^ R package. Finally, we obtained 19 clusters (resolution = 0.3) (Supplementary Fig. S1c). Two clusters (15 and 18) were excluded because they contained fewer than 100 cells or did not express known markers, and 38,097 cells were retained for the subsequent analysis. Differential gene analysis was performed by the MAST method^88^ (“FindAllMarkers” function) and the donor as the latent variable. DEGs were identified with Bonferroni-adjusted *P* values smaller than 0.05 and log_2_-fold-change values larger than 0.25. The second round of clustering procedures for CD8^+^ T and macrophage cells was the same as above: starting from normalized expression matrix with SCTransfrom method, performing integration analysis with Harmony method, and clustering with “FindNeighbors” and “FindClusters” function.

### scATAC-seq data processing

Raw sequencing data were processed with Cell Ranger ATAC (v1.2.0, 10× Genomics) software for demultiplexing, aligning to GRCh38 human reference genome, and generating peak-barcode matrices. Signac (v1.2.1)^89^ R package was used to perform subsequent analysis. Low-quality cells were removed based on the following criteria: peak region fragments > 1000, peak region fragments < 20,000, reads in peaks > 15%, blacklist ratio < 0.05, nucleosome signal < 4 and TSS enrichment > 3. After filtering, a dataset from three samples comprising 88,392 peaks from 24,173 cells remained for latent semantic indexing (LSI) analysis. Clustering and dimensionality reduction were then performed on the corrected LSI components by the Harmony^86^ method. Finally, we obtained 15 clusters with resolution = 0.5 and dims = 2:15 (Supplementary Fig. S1e). The activity of each gene was quantified by examining the local chromatin accessibility, including the 2 kb upstream of the transcriptional start site and gene body. The accessible chromatin peaks for each cell type were identified by the MACS2 method^23^. Differential chromatin accessibility analysis was performed by the “FindAllMarkers” function. Differentially accessible chromatin regions were identified with Bonferroni-adjusted *P* values smaller than 0.05 and log_2_-fold-change values larger than 0.25. The genomic regions containing accessible chromatin peaks were annotated by ChIPSeeker (v1.26.2)^90^ with the UCSC database on hg38^91^. A similar analysis strategy as described above was used to investigate the subpopulation for CD8^+^ T cells and macrophages.

### Integrated analysis of scRNA-seq and scATAC-seq data

To help to interpret the scATAC-seq data, we applied Seurat’s integration framework to identify the pairs of corresponding cells between two modalities data. The shared correlation patterns between scATAC-seq gene activity and scRNA-seq gene expression were identified by the “FindTransferAnchors” function (reduction = ‘cca’). Then the cell type label of each cell in scATAC-seq data was predicted by “TransferData” function (weight.reduction = ‘lsi’ and dim = 2:15). A total of 21,272 cells were left after filtering using a maximum prediction score ≥ 0.5 (Supplementary Fig. S1f). The filtered scATAC-seq object was reprocessed with LSI, batch corrected with Harmony algorithm, and clustered with SLM algorithm. The Jaccard index was used to assess the consistency between cell identities predicted by label transfer and curated annotations based on gene activities of known markers (Supplementary Fig. S1i).

### Single-cell copy number variation analysis

The inferCNV (v1.6.0) (inferCNV of the Trinity CTAT Project, https://github.com/broadinstitute/inferCNV) R package was used to distinguish malignant cells by inferring chromosomal CNVs based on the gene expression data. The CD8^+^ T cells and NK/NKT cells as normal reference cells were used to estimate CNVs for the potential tumor cell population. A gene ordering file from the human GRCh38 assembly containing each gene’s chromosomal start and end positions was created as the input of the “gene_order_file” parameter. The raw count matrix and annotation file were input to run inferCNV with cutoff = 0.1.

### Single-cell transcription factor activity analysis

Single-cell TF motif activity was estimated for a set of 870 TFs from the Catalog of Inferred Sequence Binding Preferences (CIS-BP) database (from chromVAR motifs ‘human_pwms_v2’) using the RunChromVAR wrapper in Signac (v1.2.1)^24,89^. Differential TF activity between cell types were calculated by “FindMarkers” function (log_2_(FC) > 1 and Bonferroni-adjusted *P* < 0.05).

### Identification of tumor-specific transcription factor

We designed a filtering strategy to identify tumor-specific TFs. For each TF, the bias-corrected deviation scores calculated by chromVAR were averaged within each cell type. Then the standard deviations (SDs) of average deviation scores were calculated across all cell types as the degree of variation. Since the distribution of SDs of all TFs was not a normal distribution or symmetric distribution, the double Median Absolute Deviation strategy was used to compute the median absolute deviation^30,92^. Candidate-specific TFs were identified with their SDs greater than the predefined threshold, which was the median of SDs plus 4× median absolute deviation. To further refine these candidate-specific TFs for tumor cells, we require that the Bonferroni-adjusted *P* value of each TF was < 0.0001, and log_2_(FC) was > 4 in tumor cells and < 1 in any other cell types. Finally, we identified 49 tumor-specific TFs.

### *Cis*-regulatory elements analysis

We identified the candidate *cis*-regulatory elements (cCREs) based on co-accessible peaks, as previously described^93^. First, the co-accessibilities between pairs of peaks were quantified by the R package Cicero (v1.3.4.11)^94^ with a graphical LASSO algorithm. The Pearson correlation as the co-accessibility score for each peak-to-peak link was computed using the “run_cicero” function with default parameters (co-access cutoff of 0.2). Next, we required one of the peaks to overlap a gene’s promoter region (distance to the transcription start site (TSS) ≤ 1 kb) and then calculated the Pearson correlation between averaged chromatin accessibility of the other peak and averaged RNA expression of this gene across all cell types. Finally, the significant gene-linked cCREs were identified with a Benjamini-Hochberg corrected *P* value < 0.05, considered as candidate cCREs.

### Transcription factor regulatory network construction

We combined the scATAC-seq data and scRNA-seq data in one cell type and identified the candidate TF target genes. For each TF, we defined the candidate target genes based on the following two criteria: (1) the promoter region of the target gene directly contains the TF binding motif; (2) the promoter region of the target gene is linked through gene-linked cCREs. The cell-type-specific TF regulatory network was constructed with these TF-gene pairs using igraph (v1.2.6) R package. Additional information, such as DEGs, was overlaid onto the network.

### Identification of drug targeting transcription factor

We used a large number of drug perturbed profiles generated from the LINCS consortium^35^ for screening candidate drugs that can target a TF. The ExperimentHub (v1.16.1)^95^ R package was used to download drug perturbed results, including a Z-score matrix from differential expression analysis of 12,328 genes for 8140 compound treatments. Only the genes with Z-score smaller than –2 were considered as differentially downregulated genes. The candidate drugs were defined as those that can significantly downregulate the expression of TF and be approved for disease treatment by the Food and Drug Administration. Finally, homoharringtonine and mitotane were selected as the candidate drugs for targeting *HOXC5*, *ISL1*, and *VENTX*, respectively (Fig. 3e).

### Identification of intratumor NMF programs

The non-negative Matrix Factorization (NMF) method was used to dissect the underlying transcriptional programs in the tumor cells from four samples and implemented by NMF (v0.23.0) R package. We applied NMF (*k* = 2:6, nrun = 30) to the relative expression matrix of tumor cells in each sample with all negative values converted to zero. The robust clustering result was determined by choosing the optimal *k* value at which the cophenetic coefficient begins to produce the maximum drop (Supplementary Fig. S4b)^36^. Finally, 11 programs were extracted among four samples (Supplementary Fig. S4c). For each program, the 30 genes with the highest NMF scores were used to score tumor cells in each sample using Seurat ‘AddModuleScore’ function. The correlations between these program scores were calculated in each sample individually. Finally, we identified two meta-programs through hierarchical clustering of averaged correlations of pairs of programs across all samples with Pearson correlation as the distance metric and Ward’s linkage (Fig. 4b). Other clustering groups were neglected because they covered less than half of the samples. The 30 genes with the highest average NMF score within each highly correlated program set were used to represent the corresponding meta-program. The clusterProfiler (v.3.18.1)^96^ R package was used to perform pathway enrichment analysis for each meta-program. The regulatory elements for genes within each meta-program were identified with the same strategy as identifying candidate TF target genes.

### Cell–cell interaction analysis

The CellPhoneDB (v2.0)^97^ was used to investigate cell–cell interaction between different cell types, especially between tumor cells and other cell types, in ccRCC. To capture interaction more systematically and comprehensively, we integrated multiple ligand–receptor resources from previous studies^98–100^ and obtained 3471 human ligand–receptor pairs (Supplementary Table S8). The gene-cell raw matrix data, cell type annotation information, and these ligand–receptor pairs were input to CellPhoneDB with threshold = 0.1. The significant ligand–receptor pairs with *P* < 0.05 and mean value ≥ 1 were selected for subsequent analyses.

### Cell culture and reagents

Human renal cell carcinoma cell lines 786-O (Cat# CRL-1932) and 769-P (Cat# CRL-1933) were obtained from American Type Culture Collection (ATCC). HEK293FT was purchased from ThermoFisher Scientific (R70007). Short Tandem Repeat (STR) analyses were performed to authenticate the identity of each Cell line used in this article. The 786-O and 769-P cells were cultured in RPMI-1640 medium (#SH30809.01; HyClone). The HEK293FT cells were cultured in Dulbecco’s Modified Eagle’s medium (#C11995599CP; Gibco). All three cell lines were supplemented with 10% fetal bovine serum (#10099-141C; Gibco), 1% penicillin-streptomycin (#SV30010; Hyclone), and 1% GlutaMaxTM Supplement (#35050-061; Gibco) and grown in a humidified 5% CO_2_ atmosphere at 37 °C.

### Vectors and lentiviral transfection

All the short hairpin RNAs (shRNA) were cloned into pLKO.1-TRC vector. Target sequences are as follows:

HOXC5#1: GCCACAGATTTACCCGTGGAT,

HOXC5#2: GCCAACAACTTGTGTCTCAAT,

VENTX#2: TAAGGAGCCAAATACCTTGCG,

VENTX#4: CATGAAACACAAACGGCAAAT,

ISL#1: GTGCGGAGTGTAATCAGTATT,

ISL#2: TCAGGTTGTACGGGATCAAAT,

OTP#1: CTATGAGCTTCACTTAATGCA,

OTP#6: CCTGTGCTCTTTCCACGCCAA.

Lentiviral particles were produced by transfecting the shRNA plasmid, packaging vectors psPAX2 and pMD2.G in HEK293FT cells at a ratio of 3:2:1 by LipoD293™ In Vitro DNA Transfection Reagent (#SL100668; SignaGen Laboratories). The media was changed after 12 h. Then 48 h after media change, the media containing the virus were collected and concentrated using the Lentivirus concentration reagent (#GM-040801-100; Genomeditech). Viral supernatants were centrifuged at 1500× *g* for 45 min and viral pellets were resuspended with RPMI-1640 medium. The lentivirus was frozen at –80 °C for further use. The efficiency of lentiviral shRNA clones was determined by real-time PCR.

### Lentiviral infection

5 × 10^5^ 786-O cells were seeded into each well of 12-well plates, at the same time, added with 200 μL of lentivirus and 10 μg/mL polybrene (#TR-1003-G; Merck Millipore). Plates were incubated at 37 °C for 12 h. The cells were then passaged and seeded into new culture dishes. Twenty-four hours after cell passage, infected lentiviral cells were selected with Puromycin (1 μg/mL, #ant-pr-1; Invivogen) for 3 days.

### Cell proliferation assay

Cell proliferation was measured over a 6-day time course using the CellTiter-Glo® Luminescent Cell Viability Assay kit (#G7572; Promega). For this assay, 500 cells were seeded into each well of a 96-well plate containing 200 μL of medium per well and cultured in normal conditions. At the time point of detection, plates were cooled down to room temperature, then 40 μL of the CellTiter-Glo reagent was added to each well. Plates were covered with aluminum foil and placed on an orbital shaker at 120 rpm for 15 min. Finally, plates were read using a microplate reader (Varioskan LUX; Thermo). All data were normalized to day 0 and presented as mean ± SD.

### RNA isolation and RT-qPCR

Total cellular RNA was isolated using TRIzol Reagent (#15596018; Thermo Fisher Scientific). The RNA concentration was measured using Nanodrop 2000 (Thermo Fisher Scientific, USA). Total RNA was reverse transcribed into cDNA using Novoscript Plus All in one First Strand cDNA Synthesis SuperMix (#E047-01S; Novoprotein) according to the manufacturer’s instructions. Quantitative reverse transcription PCR was performed per the manufacturer’s protocol on the Bio-RAD CFX Connect^TM^ system using SYBR Green Master Mix (#E096-01; Novoprotein) and gene-specific primers. The qPCR primers sequences are as follows:

HOXC5-fwd: 5′-AGAGCCCCAATATCCCTGC-3′,

HOXC5-rev: 5′-CGGTGGGAAAGTGATGCTT-3′,

VENTX-fwd: 5′-CCGTCAGCATCAAGGAGG-3′,

VENTX-rev: 5′-CTGGACCTCTGAGAGCTGC-3′,

ISL1-fwd: 5′-TACGGGATCAAATGCGCCAA-3′,

ISL1-rev: 5′-CACACAGCGGAAACACTCGAT-3′,

OTP-fwd: 5′-GCACAGCTCAACGAGTTGGA-3′,

OTP-rev: 5′-GTCAGCCCGATACGCAGTG-3′.

### Drug treatment

For cell proliferation assay, 500 cells of 786-O or 769-P were seeded into 96-well plates containing 200 μL of medium per well. After 24 h of culture at 5% CO_2_ at 37 °C, cells were exposed to 50 nM homoharringtonine (#HY-14944; MedChemExpress) for 2 days or 30 μM mitotane (#S1732; Selleckchem) for 3 days. Every 24 h, cell proliferation was measured using the CellTiter-Glo® Luminescent Cell Viability Assay kit (#G7572; Promega). For detecting the mRNA level of the targeted gene induced by drug treatment, 1 × 10^5^ 786-O or 769-P cells were seeded into each 60 mm cell culture dish. After 24 h of culture at 5% CO_2_ at 37 °C, cells were treated with 50 nM homoharringtonine (HHT) for 2 days or treated with 30 μM mitotane for 3 days. Then cells were harvested for detecting the mRNA level of the targeted genes.

### Apoptosis assay

Cells were collected, then washed twice with cold PBS. After that, cells were resuspended with binding buffer and stained with Annexin V-FITC (# 40302ES50; YEASEN) for 15 min in the dark at room temperature. After incubation, cell apoptosis analysis was measured by flow cytometer (Beckman, CytoFLEX LX). Data were analyzed with FlowJo V10 software.

### Protein extraction and western blotting

Cells were collected, washed with PBS, and lysed in RIPA Lysis Buffer (#P0013C; Beyotime) with protease inhibitor cocktail (#04693132001; Roche). The lysates were incubated on ice for 15 min and cleared by centrifugation at 12,000× *g* at 4 °C for 15 min. Protein concentration was determined using the Bradford protein assay kit (#P0006C; Beyotime). The lysates were mixed with loading buffer (#E151-05; Genstar) and boiled for 10 min. Samples were loaded onto 10% 15-well sodium dodecyl sulfate-polyacrylamide gel electrophoresis, electrophoresed, and then transferred to 0.45-μm PVDF membranes (#IPVH00010; Merckmillipor). Blocking was performed for 1 h with 5% non-fat milk (#A600669-0250; Sangon Biotech) in TBST, and blotting was performed with primary antibodies at 4 °C overnight, followed by secondary antibodies. Finally, the membrane was incubated with an enhanced chemiluminescence ECL (#34580; Thermo Scientific), and the images were obtained by using ChemiDoc XRS + Imaging System (#1708265; BIO-RAD). The following antibodies were used: monoclonal anti-GAPDH (#60004-1 Ig,1:20,000; proteintech), polyclonal anti-PARP (#9532, 1:1000; Cell Signaling Technology), anti-rabbit IgG (#7074, 1:3000; Cell Signaling Technology), and anti-mouse IgG (#7074, 1:5000; Cell Signaling Technology).

### Tumor xenograft model

For tumorigenicity assay in vivo, six-week-old NPSG mice were randomly divided into shNT, shHOXC5#1, shHOXC5#2, shISL1#1, and shISL1#2 groups, five mice per group. 5 × 10^6^ 786-O cells mixed with equal volume Matrigel (#354277; Corning) were injected subcutaneously into the right-back of NPSG mice. After implantation for 70 days, mice were sacrificed and the xenograft tumors were removed. The tumor volume was calculated by this formula: volume(mm^3^) = (longer diameter × shorter diameter^2^)/2.

### Public data acquisition

Normalized counts and clinical information from TCGA RNA-seq data of kidney renal clear cell carcinoma were downloaded from the Broad Institute GDAC Firehose constitutes (https://gdac.broadinstitute.org/). The normalized expression data of anti-PD-1 (Nivolumab) treatment for ccRCC (Checkmate025) was obtained from the previous study^48^. The enrichment of TF motifs for 18 tumor tissue types was downloaded from the supplementary material of the original article^34^. Three additional scRNA-seq datasets of ccRCC were obtained from the data materials provided by the authors^11,13,14^.

### Statistical analysis

Gene Set Enrichment Analysis (GSEA) for DEGs was performed by the GSEA desktop application (v4.1.0)^101^. Enrichment analysis for differentially accessible chromatin regions was performed by rGREAT (v1.22.0)^102^ R package. The VISION (v2.0.0)^44^ R package was used to calculate the signature score of the immune gene sets for a single cell. Overall survival analyses were performed by the Log-rank test, and *P* < 0.05 are considered significant. Details of all statistical tests used can be found in the corresponding figure legends.

## Supplementary information

Supplementary Figures S1-S7

Supplementary Table S1

Supplementary Table S2

Supplementary Table S3

Supplementary Table S4

Supplementary Table S5

Supplementary Table S6

Supplementary Table S7

Supplementary Table S8

## Data Availability

All the multi-omics raw sequencing data have been deposited into the National Center for Biotechnology Information Sequence Read Archive under accession number PRJNA768891. The custom code used for this paper is available on GitHub (https://github.com/Dragonlongzhilin/RenalTumor).
