# Supplementary material for: Single-cell multiomics analysis reveals regulatory programs in clear cell renal cell carcinoma
Source: Cell Discov. 2022 Jul 19;8:68. doi: 10.1038/s41421-022-00415-0 (PMC9296597; doi:10.1038/s41421-022-00415-0)
Supplement: Supplementary file 1 — Supplementary Figures S1-S7 [file 41421_2022_415_MOESM1_ESM.pdf]

Supplementary Figure 1

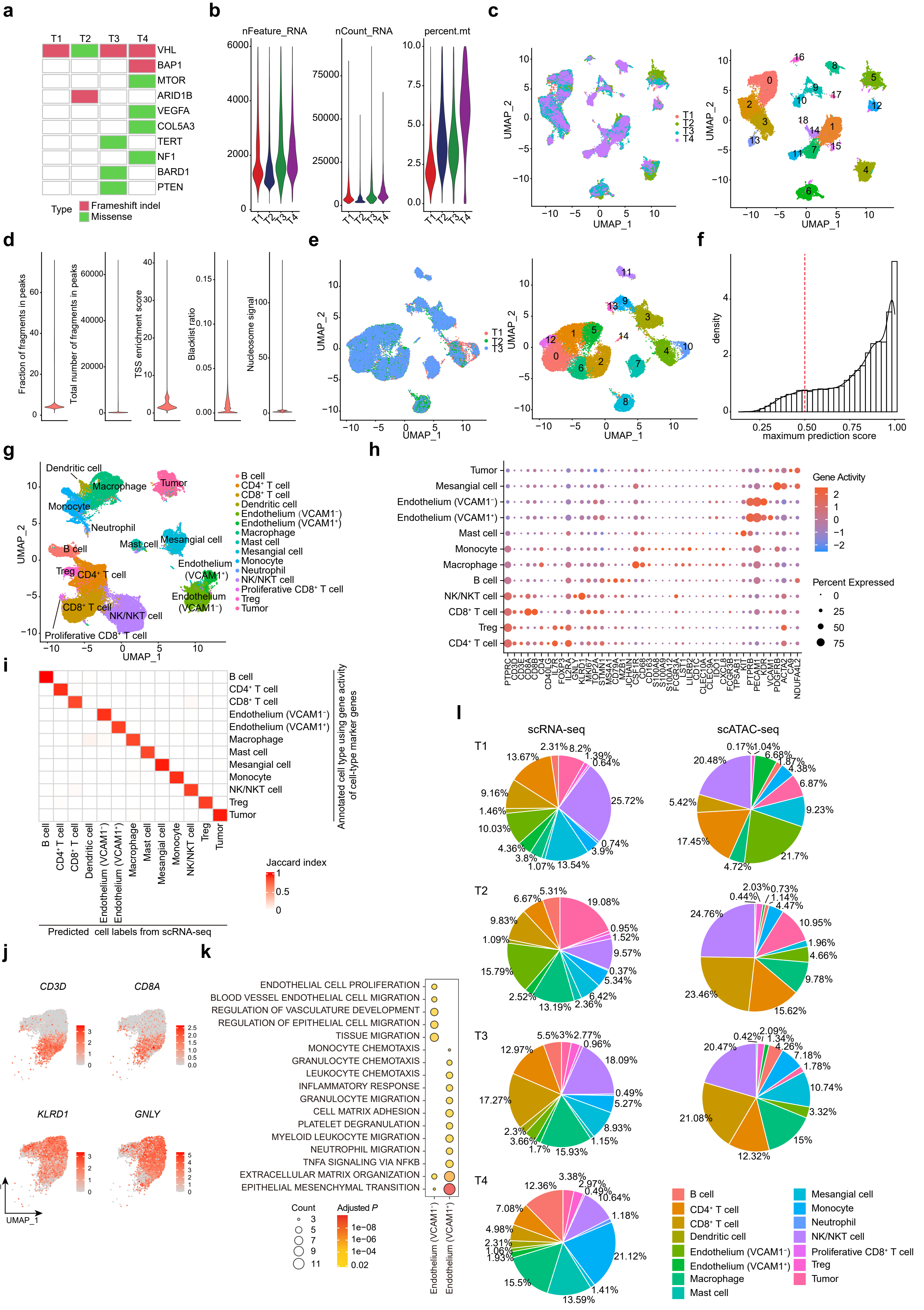

Supplementary Figure 1. Quality control and integration of scRNA-seq and scATAC-seq profiles for ccRCC. **a** Heatmap of the distribution of mutation events within four samples. **b** Violin plots showing quality control metrics of scRNA-seq data for high-quality cells in four samples. **c** UMAP of 38,600 high-quality cells was captured from the scRNA-seq data, colored by the patient (left) and cluster information (right). **d** Violin plots showing quality control metrics of scATAC-seq data for high-quality cells in three samples. **e** UMAP of 24,173 high-quality cells was captured from the scATAC-seq data, colored by the patient (left) and cluster information (right). **f** Distribution of maximum prediction scores of scATAC-seq cells calculated by the label transfer algorithm of the Signac package. The scATAC-seq dataset was filtered using a 50% prediction score threshold for cell-type assignment. **g** UMAP plot showing the coembedding of scATAC-seq data and scRNA-seq data with transfer labels. **h** Dot plot showing the gene activity of canonical marker genes. **i** Heatmap of Jaccard similarity for cell-type annotated strategies using label transfer and gene activity. **j** UMAP feature plots representation of the expression of $CD8^+$ T and NK cell markers in NK/NKT population. **k** Dot plot showing significantly enriched pathways for differential expressed genes (adjusted $P < 0.05$ and $\log_2(FC) > 1$) in each endothelium subpopulation. **l** Pie charts of the proportions of cell types in each sample.

Supplementary Figure 2

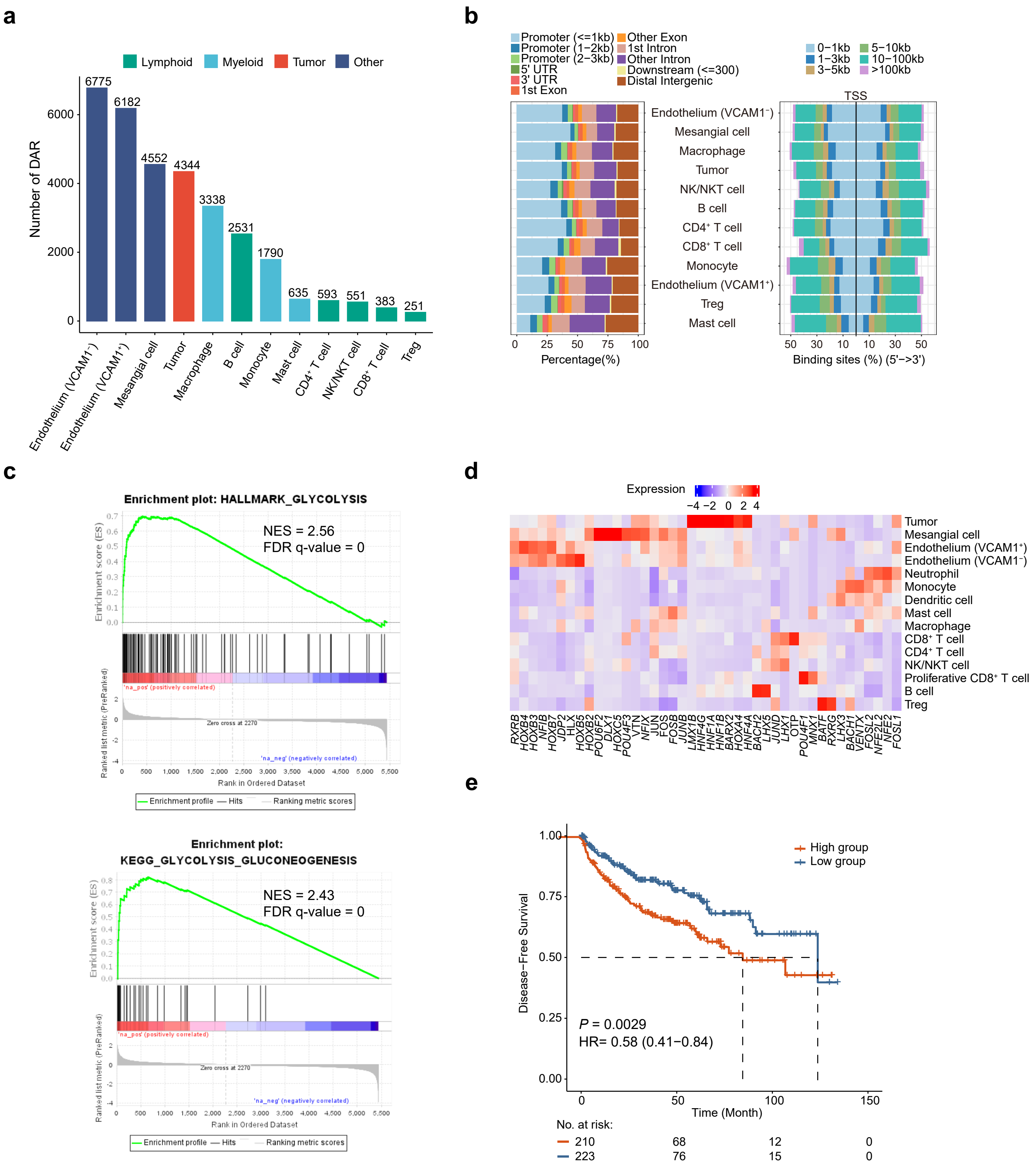

Supplementary Figure 2. Characterization of DARs within ccRCC. a Bar plot showing the number of DARs for each cell type. **b** Bar plots showing the annotated genomic locations (left) and distance of the transcription start site (right) of DARs for each cell type. **c** Gene set enrichment analysis (GSEA) for significantly upregulated genes ($\log_2(FC) \geq 0.25$ and adjusted $P < 0.05$) in tumor cells. **d** Heatmap showing the scaled log-normalized expression for tumor-specific TFs in the scRNA-seq dataset. **e** The Kaplan–Meier disease-free survival curves of TCGA-KIRC patients grouped by the average expression of tumor-specific TFs (with the median value as the threshold). HR, hazard ratio.

Supplementary Figure 3

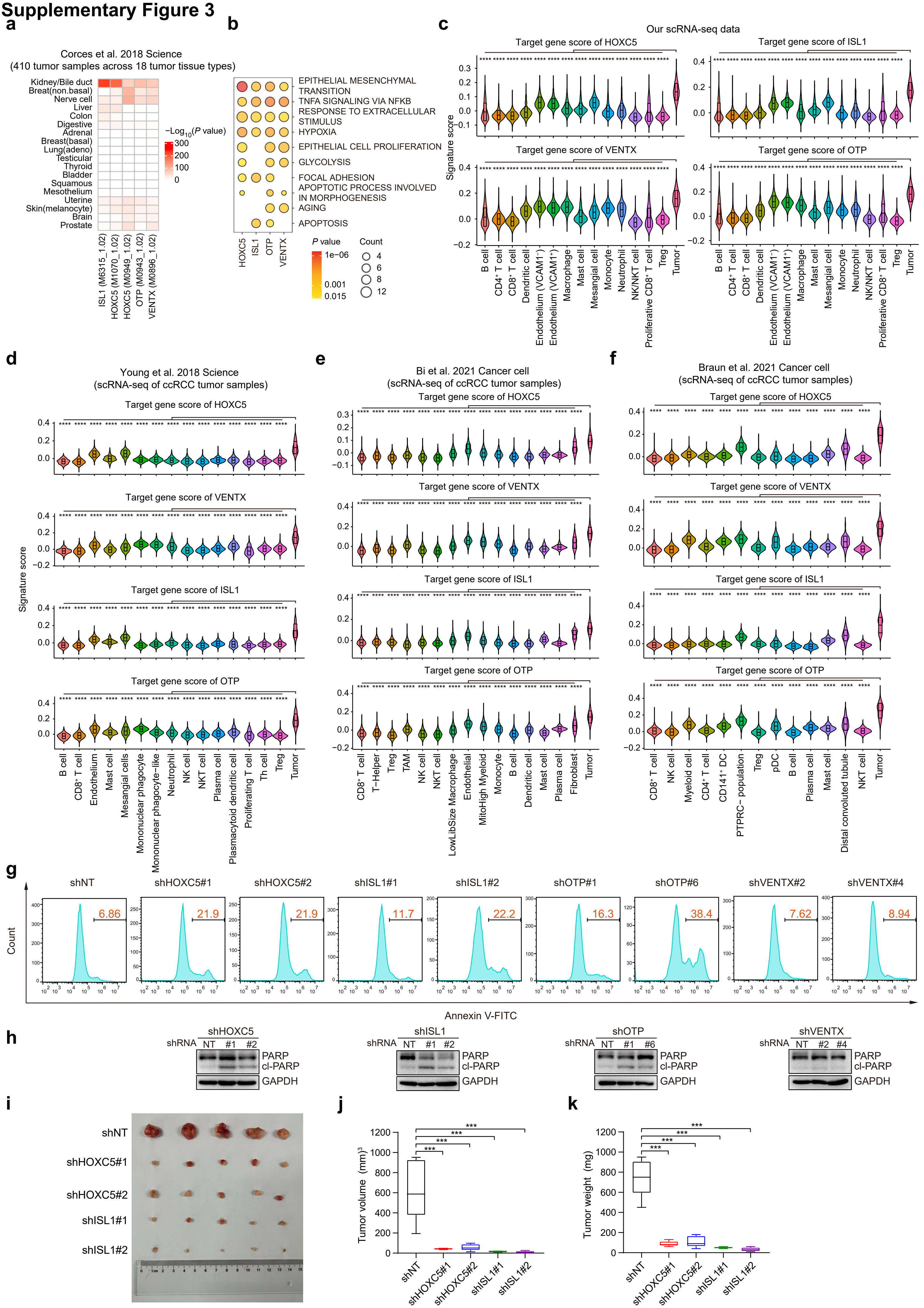

Supplementary Figure 3. Characterization of the four TFs in tumor cells. a Enrichment of four TFs in specific peak sets from each of the 18 tumor tissue types defined by Corces et al.. The significance of the motif's representation within the cluster-specific peaks compared to the pan-cancer peak set was determined by a hypergeometric test. **b** Dot plot showing significantly enriched pathways for targeted genes of each TF. **c**-**f** Violin plots of signature scores for the targeted genes of four TFs in the scRNA-seq datasets of ccRCC tumors from this study (c), Young et al. (d), Bi et al. (e), and Braun et al. (f). The Seurat's AddModuleScore function was used to calculate the signature score. **g** Apoptosis rates of the 786-O cells after *HOXC5*, *ISL1*, *OTP*, or *VENTX* knockdown were examined by flow cytometry (Annexin V-FITC staining approach). **h** The cleavage of PARP was detected by Western blotting following *HOXC5*, *ISL1*, *OTP*, or *VENTX* knockdown in the 786-O cell line. **i** Photograph of the subcutaneous tumor resected from NPSG mice xenografts 70 days after implantation with 786-O cell line transfected with shNT or shHOXC5s or shISL1s. **j**, **k** Tumor volume (j) and weight (k) were measured. Data are presented as mean ± SD. *** $P < 0.001$, n = 5.

Supplementary Figure 4

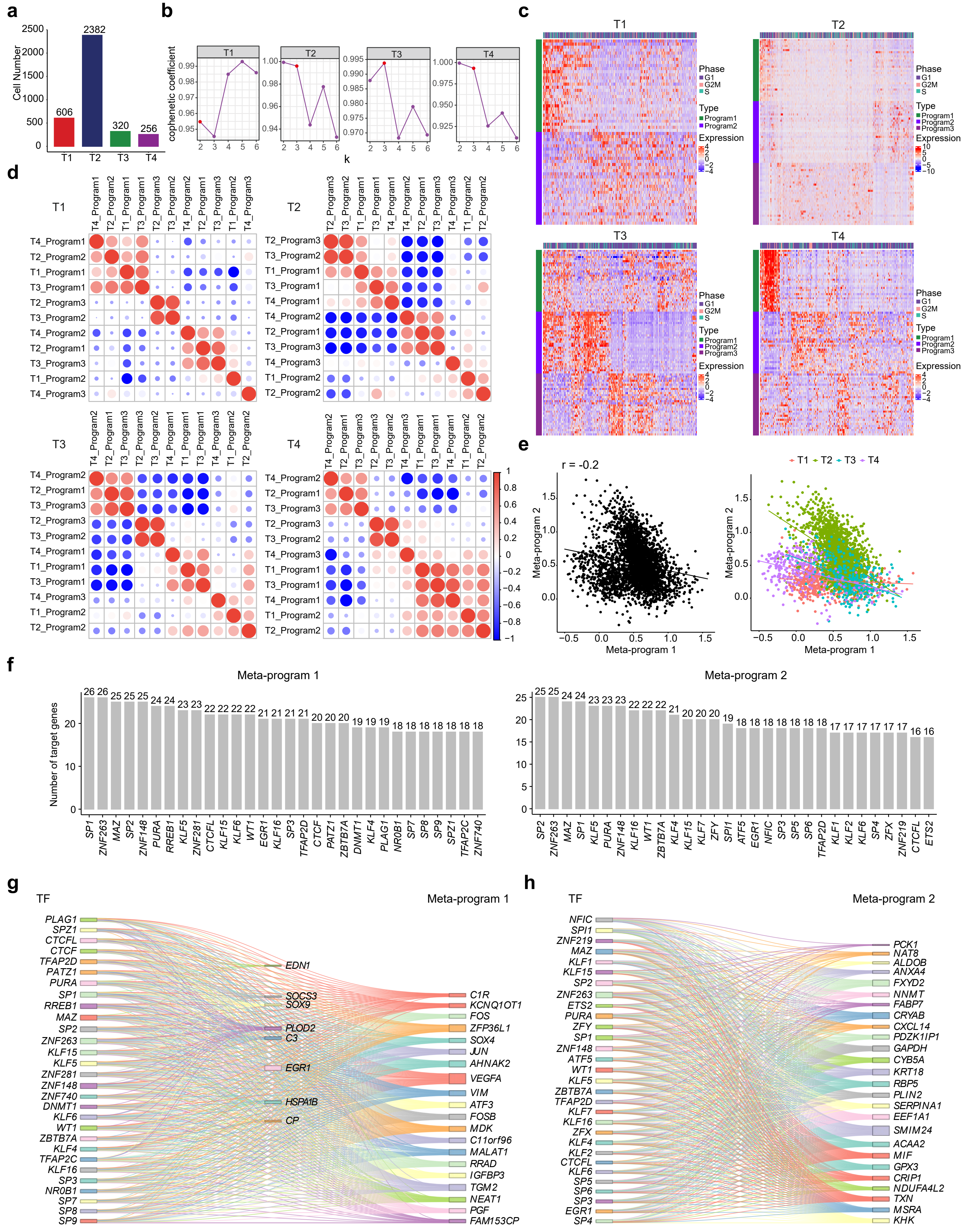

Supplementary Figure 4. Malignant transcriptional programs in ccRCC. **a** Bar plot showing the number of tumor cells in each sample. **b** Line plots showing cophenetic measure changes in each sample individually. The abscissa corresponding to the red dot on each plot indicates the optimal k value for NMF. **c** Heatmaps of relative expression levels for genes of each program in four samples individually. **d** Dot plots showing the Pearson correlation between pairs of programs in each sample individually. **e** Scatter plots showing the correlation between meta-program 1 and meta-program 2 across all samples (left) and in each sample (right), respectively. **f** The top 30 TFs with the highest number of regulatory relationships with meta-program 1 (left) and meta-program 2 (right), respectively. **g, h** Sankey diagrams showing the regulatory relationship between the top 30 TFs and genes within meta-program 1 and meta-program 2.

Supplementary Figure 5

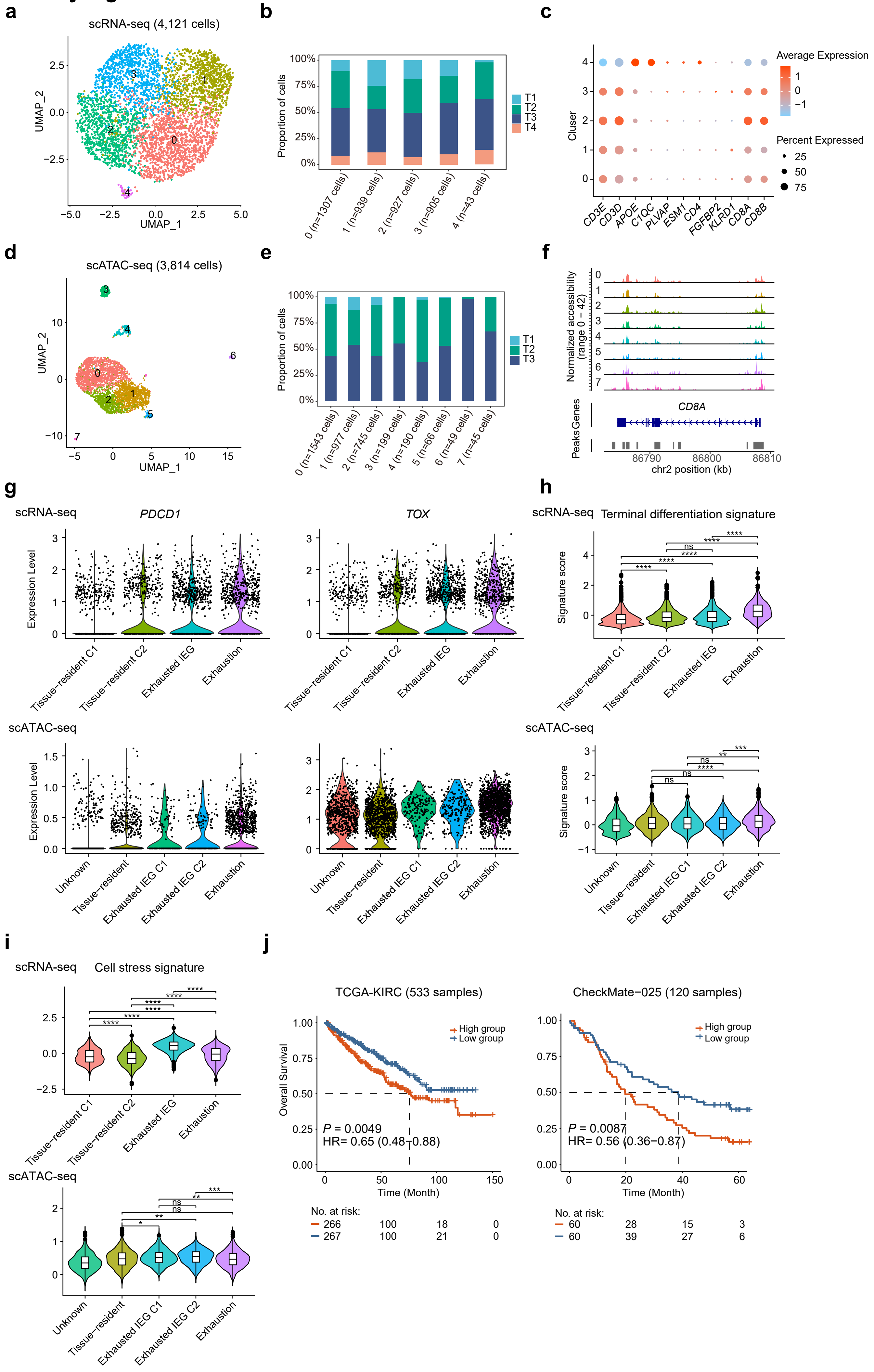

Supplementary Figure 5. Characterization of CD8⁺ T cells in ccRCC. **a** UMAP of 4,121 $CD8^+$ T cells captured from the scRNA-seq data, colored by clustering information. **b** Bar plot of the proportion of $CD8^+$ T cluster by sample in the scRNA-seq dataset. **c** Dot plot showing marker genes expression in each $CD8^+$ T cluster. **d** UMAP of 3,814 $CD8^+$ T cells captured from the scATAC-seq data, colored by clustering information. **e** Bar plot of the proportion of $CD8^+$ T cluster by sample in the scATAC-seq dataset. **f** Genome tracks of aggregated scATAC-seq data in the CD8A gene locus in each cluster. **g** Violin plots of normalized gene expression for *PDCD1* and *TOX* in each $CD8^+$ T cell cluster of scRNA-seq (top) and scATAC-seq data (bottom), respectively. **h**, **i** Violin plots showing the cell stress and terminal differentiation signature scores for each $CD8^+$ T cell cluster in the scRNA-seq (top) and scATAC-seq dataset (bottom). The signature score for each cell was calculated by the VISION method. Two-sided Wilcoxon test. ns, no significance; * $P < 0.05$; ** $P < 0.01$; *** $P < 0.001$; **** $P < 0.0001$. **j** The Kaplan–Meier overall survival curves of TCGA-KIRC and CheckMate-025 patients grouped by the averaged expression of DEGs in $CD8^+$ exhaustion cluster in the scRNA-seq dataset (with the median as the threshold). The *P* value was calculated by the log-rank test. HR, hazard ratio.

Supplementary Figure 6

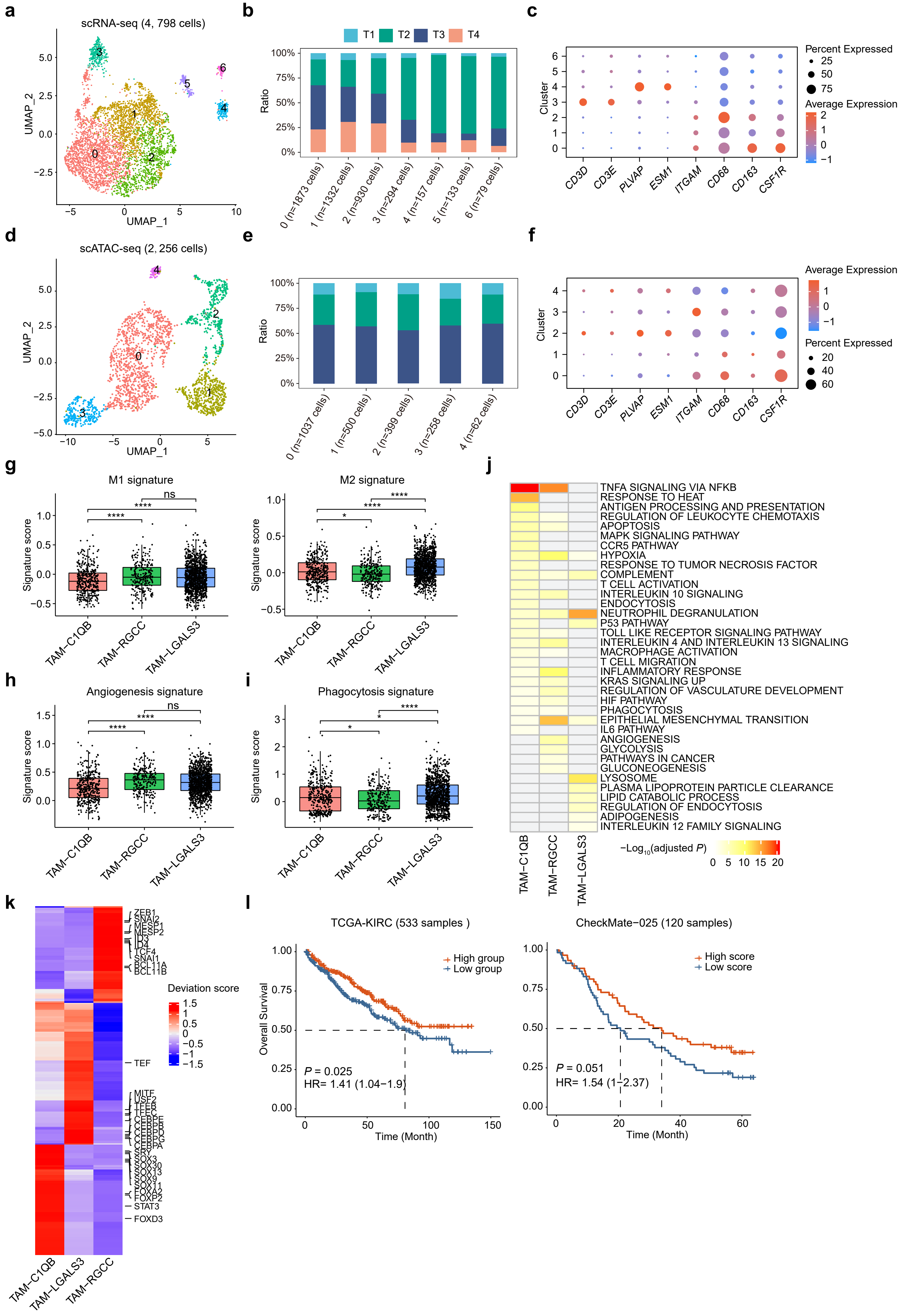

Supplementary Figure 6. Characterization of TAMs in ccRCC. a UMAP of 4,798 TAMs captured from the scRNA-seq data, colored by clustering information. **b** Bar plot of the proportion of TAM cluster by sample in the scRNA-seq dataset. **c** Dot plot showing gene expression patterns for the marker genes in each TAM cluster. **d** UMAP of 2,256 TAMs captured from the scATAC-seq data, colored by clustering information. **e** Bar plot of the proportion of TAM cluster by sample in the scATAC-seq data. **f** Dot plot showing activity patterns for the marker genes in each TAM cluster. **g** Violin plots showing the M1 (left) and M2 (right) signature scores for each TAM cluster in the scATAC-seq data. The signature score was calculated by the VISION method. Two-sided Wilcoxon test. ns, no significance; * $P < 0.05$; ** $P < 0.01$; *** $P < 0.001$; **** $P < 0.0001$. **h**, **i** Violin plots showing the angiogenesis and phagocytosis signature scores for each TAM cluster in the scATAC-seq data. The signature score was calculated by the VISION method. Two-sided Wilcoxon test. ns, no significance; * $P < 0.05$; ** $P < 0.01$; *** $P < 0.001$; **** $P < 0.0001$. **j** Heatmap of significantly enriched pathways for each TAM cluster in the scRNA-seq dataset. The redder, the smaller the adjusted *P* value. **k** Heatmap of scATAC-seq chromVAR bias-corrected deviation scores for the significantly different TFs in each TAM cluster. The top 10 TFs of each cluster are indicated to the right of the plot. **l** The Kaplan–Meier overall survival curves of TCGA-KIRC and CheckMate-025 patients grouped by the signature score for candidate target genes of *MEF2C* (with the median as the threshold). The *P* value was calculated by the log-rank test. HR, hazard ratio.

Supplementary Figure 7

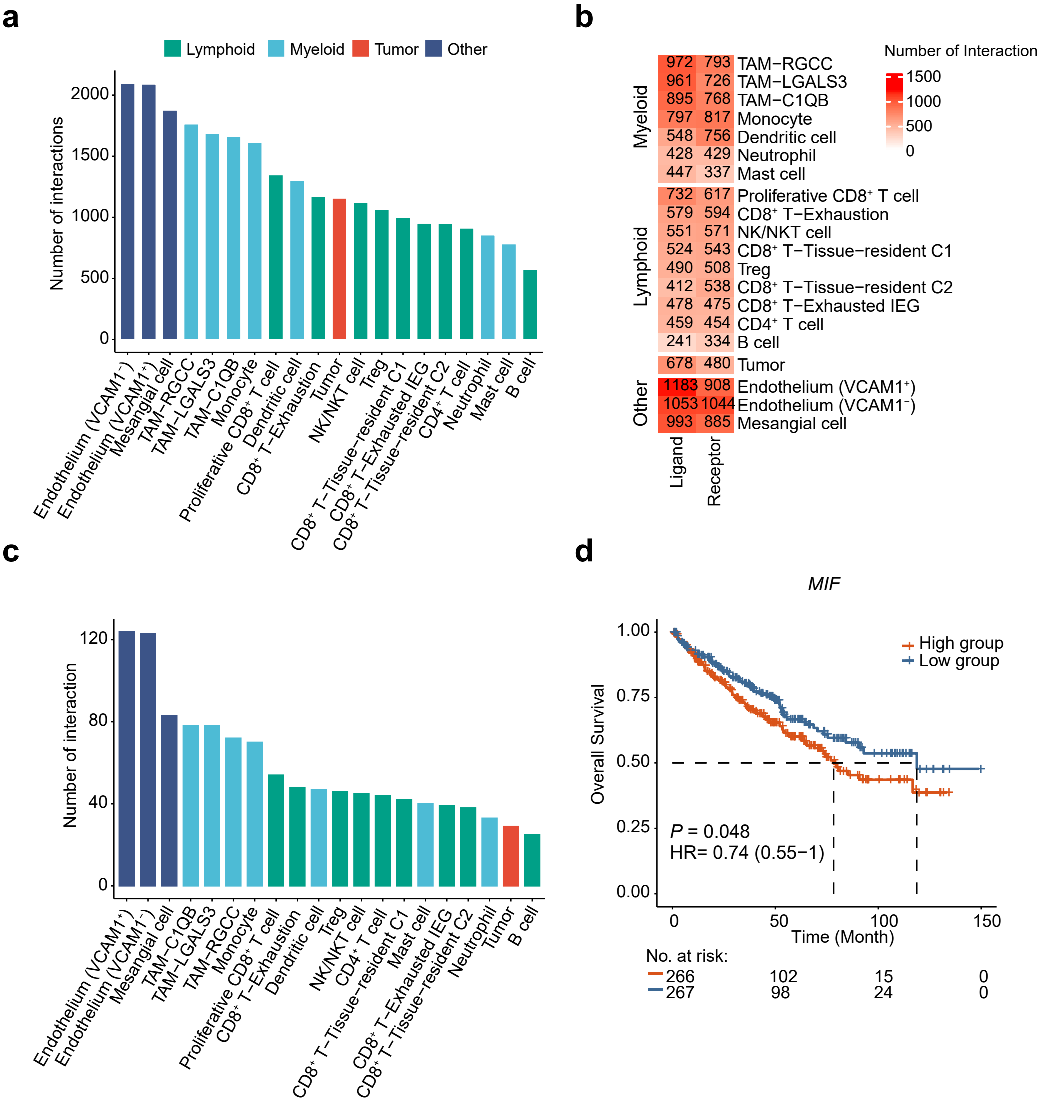

Supplementary Figure 7. Ligand–receptor-based interaction in ccRCC microenvironment. **a** Bar plot showing the number of significant ligand–receptor interactions for each cell type. **b** Heatmap of the number of ligands and receptors involved in significant ligand–receptor pairs. **c** Bar plot showing the number of significant ligand–receptor interactions between tumor cells and other cell types. **d** The Kaplan–Meier overall survival curves of TCGA-KIRC patients grouped by the expression of *MIF* (with the median as the threshold).
